# A selectable all-in-one CRISPR prime editing piggyBac transposon allows for highly efficient gene editing in human cell lines

**DOI:** 10.1038/s41598-021-01689-2

**Published:** 2021-11-12

**Authors:** Reto Eggenschwiler, Thomas Gschwendtberger, Christian Felski, Christopher Jahn, Florian Langer, Jared Sterneckert, Andreas Hermann, Jonathan Lühmann, Doris Steinemann, Alexandra Haase, Ulrich Martin, Susanne Petri, Tobias Cantz

**Affiliations:** 1 Research Group Translational Hepatology and Stem Cell Biology, Department of Gastroenterology, Hepatology and Endocrinology, Hannover Medical School, 30625 Hannover, Germany; 2 REBIRTH-Research Center for Translational Regenerative Medicine, Hannover Medical School, 30625 Hannover, Germany; 3 Department of Neurology, Hannover Medical School, 30625 Hannover, Germany; 4 Center for Systems Neuroscience, Hannover Medical School, 30625 Hannover, Germany; 5 Center for Regenerative Therapies Dresden (CRTD), Technische Universität Dresden, 01307 Dresden, Germany; 6 Translational Neurodegeneration Section “Albrecht-Kossel”, Department of Neurology and Center for Transdisciplinary Neurosciences Rostock (CTNR), University Medical Center Rostock, University of Rostock, Rostock, Germany; 7 German Center for Neurodegenerative Diseases (DZNE) Rostock/Greifswald, 18147 Rostock, Germany; 8 Institute of Human Genetics, Hannover Medical School, 30625 Hannover, Germany; 9 Leibniz Research Laboratories for Biotechnology and Artificial Organs (LEBAO), Department of Cardiothoracic, Transplantation and Vascular Surgery, Hannover Medical School, 30625 Hannover, Germany; 10 Biomedical Research in Endstage and Obstructive Lung Disease (BREATH), German Center for Lung Research (DZL), 30625 Hannover, Germany; 11 Max Planck Institute for Molecular Biomedicine, Cell and Developmental Biology, 48149 Munster, Germany

**Keywords:** Genetic engineering, Amyotrophic lateral sclerosis

## Abstract

CRISPR prime-editors are emergent tools for genome editing and offer a versatile alternative approach to HDR-based genome engineering or DNA base-editors. However, sufficient prime-editor expression levels and availability of optimized transfection protocols may affect editing efficiencies, especially in hard-to-transfect cells like hiPSC. Here, we show that piggyBac prime-editing (PB-PE) allows for sustained expression of prime-editors. We demonstrate proof-of-concept for PB-PE in a newly designed lentiviral traffic light reporter, which allows for estimation of gene correction and defective editing resulting in indels, based on expression of two different fluorophores. PB-PE can prime-edit more than 50% of hiPSC cells after antibiotic selection. We also show that improper design of pegRNA cannot simply be overcome by extended expression, but PB-PE allows for estimation of effectiveness of selected pegRNAs after few days of cultivation time. Finally, we implemented PB-PE for efficient editing of an amyotrophic lateral sclerosis-associated mutation in the SOD1-gene of patient-derived hiPSC. Progress of genome editing can be monitored by Sanger-sequencing, whereas PB-PE vectors can be removed after editing and excised cells can be enriched by fialuridine selection. Together, we present an efficient prime-editing toolbox, which can be robustly used in a variety of cell lines even when non-optimized transfection-protocols are applied.

## Introduction

**Figure 1:**
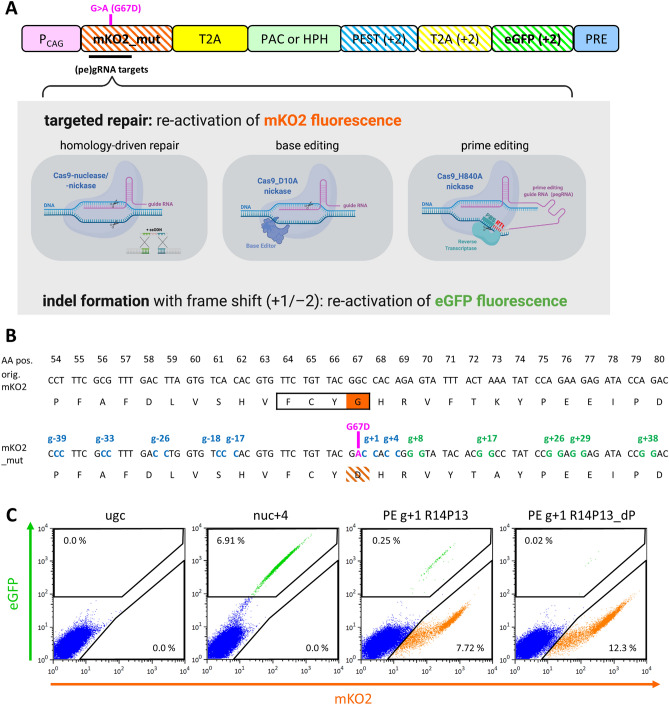
A traffic light reporter allows for concurrent estimation of gene correction and indel formation in human cells. (**A**) Schematic overview of TLR, P_CAG_: CMV i/e enhancer—chicken β-actin promoter—rabbit globin intron (CAG) promoter; mK02_mut: a variation of the monomeric Kusabira Orange 2 gene with a G67D point mutation in the fluorochrome; PAC: puromycin N-acetyltransferase; HPH: hygromycin-B-phosphotransferase. Graphics in gray shaded area were created with Biorender.com. (**B**) Detailed view of mK02_mut sequence compared to original mK02. PAM sites for regular SpCas9 CRISPR sgRNAs are depicted in blue (3′ → 5′) and green (5′ → 3′). Amino acids at positions 71 and 73 were modified to allow for efficient ABE targeting. FCYG fluorochrome is framed in black and fluorescence-aborting G67D point mutation is depicted in pink. (**C**) Example FACS plots depicting 293-TLR cells transfected with Cas9 nuclease and g+4 gRNA or with prime editor and g+1 R14P13 or g+1 R14P13_dP (PAM disrupting) pegRNA. Cells transfected with Cas9 nuclease and an empty sgRNA vector served as unguided control (ugc).

Addition of CRISPR prime editors (PEs) to the repertoire of tools for directed genome editing has opened new avenues in life sciences and biomedical applications. Previous CRISPR/Cas9 approaches that were based on the repair of Cas9-induced DNA double strand breaks via non-homologous end joining (NHEJ) or homology driven-repair (HDR) exhibit limitations with respect to undesired off-target DNA-cleavage and overall DNA editing efficacies. Another approach is utilizing engineered Cas-proteins that allow for direct DNA editing as base editors (BEs) by mediating highly efficient transition of purine (A ↔ G) or pyrimidine (C ↔ T) bases with low rates of unintended indel formation. However, BEs have certain PAM-distal editing ranges, and undesired co-editing of neighboring bases is possible^1^. The recently developed prime editing (PE) approach allows the introduction of transversion mutations as well as introduction or deletion of defined DNA stretches in specific genomic loci^2^. Editing efficiencies as well as rates of introduction of undesired indels by PE-vectors are as favorable as for DNA base editors (BEs), but, in contrast, PEs can generally be employed for targeted editing of a long range upstream of the PE-induced DNA nick. In the case of SpCas9_H840A constructs this applies for the PAM -3 nt proximal region. Moreover, PE-based genome editing techniques generally offer the possibility of co-editing the PAM site (PAM disruption) in order to avoid re-editing of already edited loci, a feature which is not present in BEs. A schematic overview of HDR-, ABE- and PE-based genome editing techniques is provided in Fig. 1A. While prime editing offers certain advantages over BE-based techniques, editing efficiencies strongly depend on design of pegRNA with respect to lengths of both, reverse transcriptase template (RTt) and primer binding site (PBS), as well as exact protospacer target^2,3^. Moreover, overall prime-editing efficiencies also depend on availability of good transfection protocols, which can be hard to establish for certain cell types, such as hiPSCs.

Here, we sought to investigate whether inadequate design of pegRNA or non-optimized transfection protocols can be overcome by extended expression of CRISPR-PE. In order to obtain fast read-out of such studies we designed a novel traffic light reporter (TLR) in which a G67D missense point mutation in the monomeric Kusabira Orange 2 (mKO2) gene abrogates fluorescence. A frame-shifted eGFP gene joined to the coding sequence allows for concurrent read-out of indel formation. We generated HEK293 and hiPSC TLR bulk cell populations by lentiviral transduction to compare different PE designs and transfection protocols. We then introduce different pegRNA designs for mKO2 gene correction into selectable piggyBac transposon-flanked CRISPR-PE cassettes, herein termed PB-PE, and analyze progress of prime editing over time. We find that PB-PE corrects more than 50% of cells with efficient pegRNAs, even when applying non-optimized transfection protocols, in both, HEK293 and hiPSC. However, constructs with inefficient pegRNAs correct less than 10% of cells even after 40 days of expression time. Finally, we implemented PB-PE in a practical-oriented manner for fast and efficient editing of an R115G point mutation in the human superoxide dismutase 1 (SOD1) gene of patient-derived hiPSC. This mutation is associated with certain cases of amyotrophic lateral sclerosis (ALS)^4^. In summary, we present PB-PE as an exciting tool for fast and simple prediction of suitable pegRNA designs as well as for more convenient application of prime editors as it is less dependent on optimization of transfection for each cell line.

## Results

### A versatile reporter vector for simultaneous analysis of CRISPR-based gene correction and indels

Simultaneous estimation of traditional, homology-based gene correction and indel formation by error-prone non-homologous end-joining (NHEJ) or related pathways can be performed using traffic light reporter (TLR) systems. In previous TLR versions, a fluorophore with a premature stop codon was joined by a 2A peptide to a frame-shifted fluorophore of a different color^5^. Here, we sought to use a missense mutation instead of a premature stop codon. Glycine at amino acid position 67 inside the FCYG fluorochrome of the monomeric Kusabira Orange 2 (mKO2) protein is highly conserved amongst known fluorophores (Supplementary Fig. 1A). We found that a G67D (GGC > GAC) missense point mutation abrogates mKO2 fluorescence (Supplementary Fig. 1B). Previously, TLR constructs have also been targeted to genomic safe harbor sites^6^. However, a broader and more random distribution of genomic reporter locations should allow for reducing locus-specific effects and be more suitable for investigation of effects across the genome. To this end, we employed lentiviral vectors, which are known to result in a semi-random integration pattern^7^. Proper functionality of a TLR bulk population requires that a large fraction of the cells can be activated and that potential dual activation resulting from cells with multiple reporter integrations is at very low level. As some artificial promoters can be prone to strong transgene silencing, especially in pluripotent stem cells (PSC), we employed the CMV i/e enhancer chicken β-actin rabbit globin-intron (CAG) promoter, which is known to promote high transgene expression at low vector copy numbers in PSC^8^. The TLR cassette consists of a for our purposes optimized version of mKO2_G67D, termed mKO2_mut, which is joined via T2A site to either puromycin *N*-acetyl-transferase (PAC) or hygromycin-B-phosphotransferase (HPH), allowing for selection of bulk populations transduced at low multiplicity of infection (MOI). In a first version of TLR, a +2 bp frame-shifted eGFP gene was directly joined to mKO2_mut-T2A-PAC, but this resulted in a considerable fraction of cells with activated eGFP (Supplementary Fig. 1C). Similar to previous TLR vector studies^5^ auxiliary start codons inside the eGFP gene were removed and placed into an upstream PEST sequence (rich in proline (P), glutamic acid (E), serine (S), and threonine (T)), which was then joined to eGFP via T2A (Fig. 1A). The mKO2 sequence in proximity of the G67D point mutation was modified to introduce NGG protospacer adjacent motifs (PAMs) for additional SpCas9 gRNAs (Fig. 1B). Of note, K73A was introduced to add a PAM for g+17 gRNA at a distance which places the G67D point mutation within the editable range of an adenine base editor (ABE)^1^. F71Y was introduced to remove a TT+Y-motif in the PAM-proximal region of g+17, which could have resulted in inefficient gRNA expression^9^. These modifications were designed based on known sequences of other fluorophore sequences, where identical amino acids were found at the respective positions (Supplementary Fig. 1A). Accordingly, we found that the modified mKO2 sequence was still encoding a brightly fluorescent protein (Fig. 1C, Supplementary Fig. 1A). HEK293 cells were transduced at MOI of 0.02 and selected using puromycin or hygromycin B, respectively, resulting in 293-TLR bulk populations. Both versions (PAC and HPH) were tested side-by-side using Cas9 nuclease or prime editors and activation was comparable (Supplementary Fig. 2J,K).

Twelve selected gRNA binding sites inside the mKO2_G67D fluorophore were employed to activate eGFP by co-transfection with a Cas9 nuclease encoding plasmid (Supplementary Fig. 2A). While relative efficiencies differed, some but not all highly active gRNAs had an ‘A’ or a ‘T’ at the PAM −4 position on the TLR target sequence (Fig. 1B, Supplementary Fig. 2A). An ‘A’ or ‘T’ at this position can frequently cause +1 nt insertions thereby additionally activating the eGFP gene^10^. Analysis of sub-cloned mKO2 sequences from eGFP+ TLR cells confirmed that seven out of fifteen eGFP-activating mutations were +1 nt insertions when using g + 4 gRNA. Meanwhile only two out of ten +1 nt insertions were detected when using g+1 gRNA, which binds just 3 nt upstream of g+4 (Supplementary Fig. 3). Ten single cell clones were established from the 293-TLR bulk population and individually tested for eGFP activation by Cas9 nuclease. While all clones were enablable, relative activation efficiencies differed. However, average activation efficiency of clonal lines was comparable to the efficiency measured in the bulk (Supplementary Fig. 2B).

**Figure 2:**
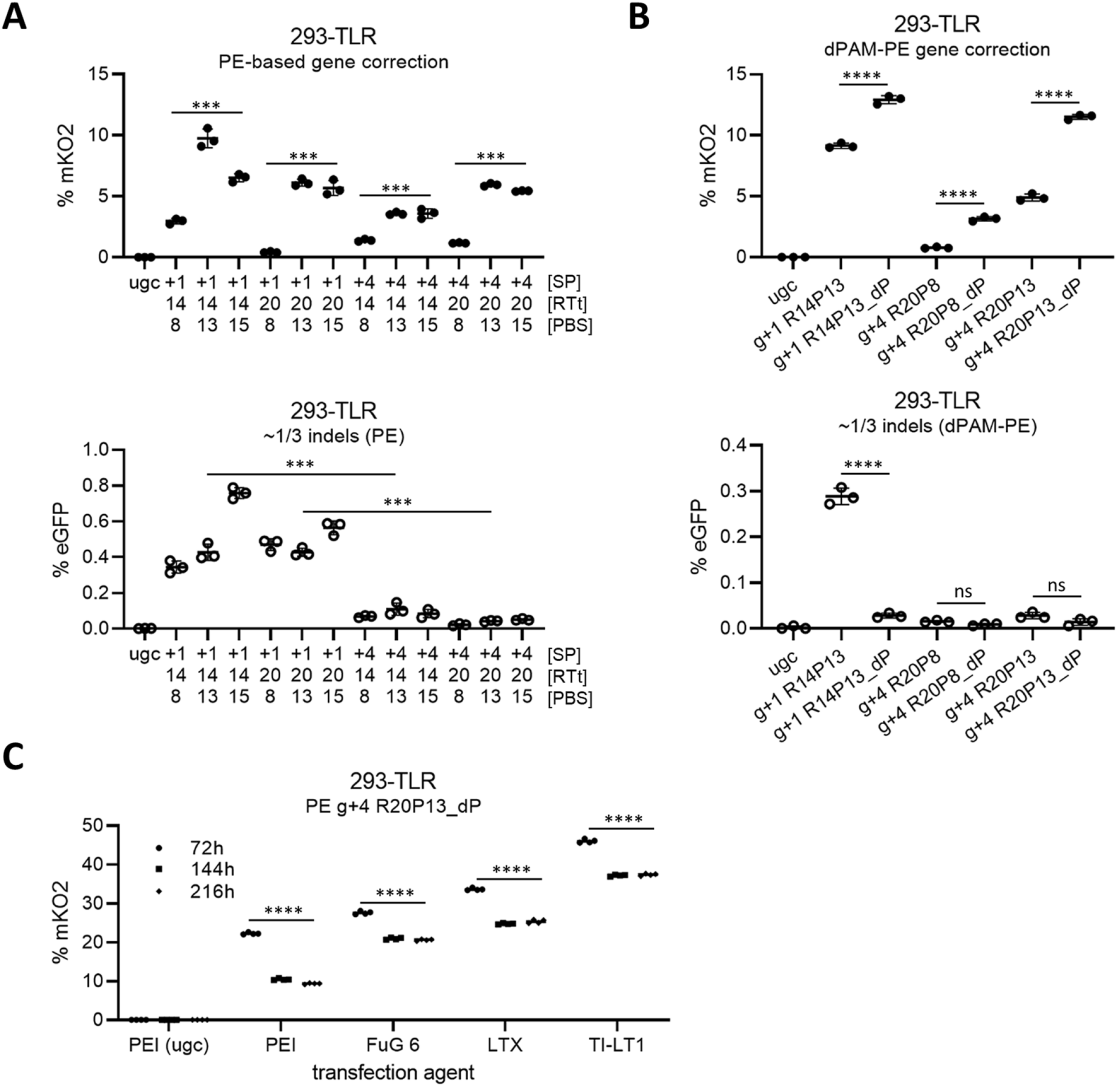
Dependence of PE activity and indel formation frequency on pegRNA design and loss of gene edited 293-TLR cells after transfection. (**A**) Gene correction and indel formation in 293-TLR cells with pCMV-PE2 prime editor and different pegRNAs. [SP] location of pegRNA protospacer; [RTt] length of pegRNA reverse transcriptase template; [PBS] length of pegRNA primer binding site. (**B**) Gene correction and indel formation of different pegRNAs with their respective counterparts harboring an additional mutation for PAM disruption (dP, dPAM) in the RTt. ugc: unguided control samples, transfected with pCMV-PE2 and an empty pegRNA vector. (**C**) Analysis of mKO2 gene corrected 293-TLR cells transfected with pCMV-PE2 and efficient g+4 R20P13_dP pegRNA using different transfection agents at different time points after transfection. Cells were split and re-analyzed every 72 h. Data are represented as ± SD from n = 3 (**A**, **B**) or n = 4 (2**C**) biological replicates and significance was calculated using 1-way ANOVA with Tukey's post-test or 2-way ANOVA with Bonferroni multiple comparisons test for data displayed as groups (****p ≤ 0.0001; ***p ≤ 0.001; ns: p > 0.05).

In order to investigate potential activation of two or even more TLR transgenes, HEK293 cells were transduced at higher MOIs of 0.1 to 5 and transfected after selection. All bulk populations could readily be activated by Cas9 nuclease as well as CRISPR-PE (Supplementary Fig. 2E). The g+1 R14P13 pegRNA was employed for this analysis, which offered high gene correction rates (9.74 ± 0.76%) but at the same time introduced a well measurable amount of indels (0.43 ± 0.04%) (Fig. 2A). Only a small fraction of double positive events (0.04 ± 0.01%) was detected, even in reporter populations originating from transduction at high MOIs (Supplementary Fig. 2E). Aiming to avoid overestimation of gene correction efficiencies, gating of flow cytometric analysis was set to count those rare events as part of eGFP rather than mKO2.

293-TLR cells were also transfected using Cas9_D10A nickase together with g+1 gRNA and ssODNs for mKO2 gene correction of 100, 150 or 200 nt. We observed best gene correction efficiencies using a 150 nt ssODN (Supplementary Fig. 2F). Employment of Cas9 nuclease instead of Cas9_D10A nickase resulted in comparable gene correction albeit at the cost of more indels (Supplementary Fig. 2G). Finally, the mKO2 signal could be activated in 293-TLR cells using two different vectors expressing the ABE7.10 adenine base editor^1^ with g+17 gRNA (Supplementary Fig. 2H). Transfection of 293-TLR cells with ABE revealed better gene correction efficiencies and very low rates of indel formation compared to ssODN-based strategies.

### Parameters influencing prime editing efficiency with transient transfection protocols in HEK-293 cells

To evaluate efficiencies of prime editors using the TLR system, prime editing gRNAs (pegRNAs) were designed to match at protospacers of g+1 and g+4, respectively. Design of pegRNA with respect to lengths of both, RTt and PBS can strongly influence PE efficiency, and best pegRNA design varies over different target loci^2^. Here, combinations of 14 or 20 nt RTt with 8, 13 or 15 nt PBS were used, which were reported to result in high gene correction efficiencies at some targets^2^. Notably, all combinations of RTt and PBS lengths resulted in detectable mKO2 activation of 293-TLR cells (Fig. 2A). Longer PBS of 13 nt offered better gene correction efficiencies than 8 nt for both, g+1 and g+4 pegRNAs, whereas extension of PBS to 15 nt didn’t further increase efficiencies for any of the pegRNA designs. Meanwhile, indel formation was more influenced by the exact binding site of the pegRNA protospacer rather than their RTt and PBS lengths, as all g+4 pegRNAs showed lower eGFP signals than g+1 pegRNAs (Fig. 2A). Co-editing of PAM sites by corresponding changes in the RTt parts of pegRNAs (dP) further improved PE gene correction efficiencies for all evaluated pegRNAs (Fig. 2B). Additionally, we observed reduced indel formation for pegRNA g+1 R14P13_dP (Fig. 2B). Additional nicking of the non-edited strand termed ‘PE3’ was previously shown to further stimulate gene correction efficiencies for some targets^2^. However, no further improvement of gene correction was observed when applying PE3 in 293-TLR cells with the most efficient pegRNAs, g+1 R14P13_dP or g+4 R20P13_dP, but some combinations caused higher indel rates (Supplementary Fig. 2C,D).

For all assays described above, 293-TLR cells were transfected using a simple polyethylenimide (PEI) protocol (which is a very cost effective transfection method) and cells were analyzed 120 h post transfection. However, mKO2 values were significantly higher when cells were analyzed at 72 h post transfection and then dropped over the following days (Fig. 2C). Theoretically, it was possible that expression of the fluorescent mKO2 transgene was resulting in a selective disadvantage for gene corrected cells. In order to investigate whether this was true or not, 293-TLR cells were transfected with ABE7.10 and g+17 gRNA and pure mKO2+ and mKO2- populations were FACS sorted and cultivated again. MKO2+ cells were then mixed 1:1 with either mKO2- sorted cells or with the original 293-TLR cell population and co-cultivated. Populations were split and FACS analyzed every 72 h. Analysis over 12 days of cultivation did not reveal a selective disadvantage or advantage of cells expressing fluorescent mKO2 (Supplementary Fig. 4A). Another possibility was that gene corrected cells were lost due to harsh transfection conditions when using PEI. To this end, the three low toxicity transfection agents Lipofectamine LTX, Fugene 6 and TransIT-LT1 were tested for PE-based gene correction using g+4 R20P13_dP pegRNA. Indeed, all tested low toxicity transfection agents resulted in higher overall mKO2 signal and lower relative amounts of corrected cells lost over the following days (Fig. 2C). In established protocols, 80,000 293-TLR cells were seeded the day before transfection. In order to further evaluate robustness of PEI transfection-based prime editing efficiency in 293-TLR cells, different amounts of cells were seeded the day before transfection. No significant difference in prime editing efficiency was detected when seeding 40,000 cells or 160,000 cells instead of 80,000 cells (Supplementary Fig. 4B,C), suggesting that the PEI transfection protocol was not very sensitive to minor differences in cell numbers.

### Parameters influencing prime editing efficiency with transient transfection protocols in hiPSC cells

**Figure 3:**
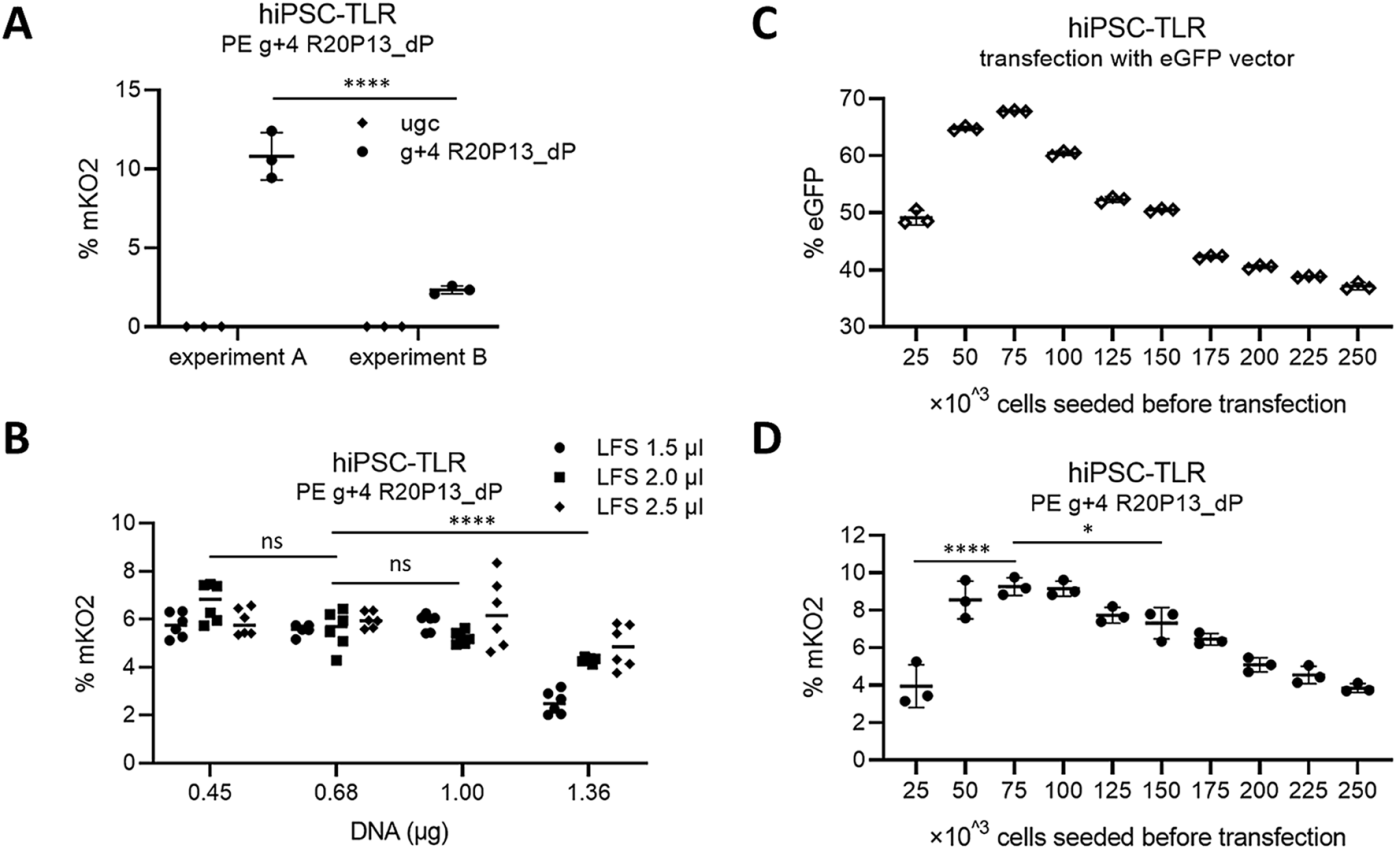
Transfection conditions can strongly influence prime editing efficiency in hiPSC. (**A**) Analysis of mK02 gene correction in two temporally separated experiments of hiPSC-TLR transfected with pCMV-PE2 prime editor and g+4 R20P13_dP pegRNA. (**B**) Tolerance of prime editing efficiency to pipetting inaccuracy in hiPSC-TLR. Cells were transfected with different volumes of Lipofectamine Stem (LFS) and different amounts of DNA per well of 12-well. (**C**, **D**) Dependency of transfection efficiency and prime editing efficiency to number of cells seeded the day before transfection. Data are represented as ± SD from n = 3 (**A**, **C**, **D**) or n = 6 (3B) and significance was calculated using 1-way ANOVA with Tukey's post-test or 2-way ANOVA with Bonferroni multiple comparisons test for data displayed as groups (****p ≤ 0.0001; *p ≤ 3.05; ns: p > 0.05).

Aiming at optimization of PE protocols for human pluripotent stem cells, TLR vector was introduced into the transgene-free phoenix hiPSC line^11^ and a reporter bulk population (hiPSC-TLR) was generated. In contrast to 293-TLR, mKO2+ cells were not lost in hiPSC-TLR after transfection with Lipofectamine Stem (LFS) transfection reagent (Supplementary Fig. 4D). However, while some attempts for PE with efficient g+4 R20P13_dP pegRNA resulted in gene correction efficiency comparable to PEI-transfected 293-TLR, other attempts resulted in considerably lower percentages of corrected cells (Fig. 3A). In order to investigate this somewhat puzzling finding, parameters which could theoretically impact transfection conditions and thus, gene correction efficiency, were examined. First, tolerance to potential pipetting errors regarding amounts of used plasmid DNA and LFS reagent was analyzed. To this end, a DNA range of 0.45–1.36 μg and a LFS range of 1.5–2.5 μl per well were examined. Measured PE efficiencies were largely indifferent in a wide range of both, DNA and LFS and only at 1.36 μg DNA, significant changes in PE efficiency were observed (Fig. 3B). Standard assays were performed using 2 μl LFS with 0.68 μg DNA per well and thus, at least twofold exceedance of DNA would be necessary in order to measure a strong impact. In light of this analysis, pipetting errors were ruled out as potential reason for observed experiment-to-experiment differences. In order to analyze whether the total number of cells per well were influencing transfection efficiency and prime editing efficiency in hiPSC-TLR, a range of 25,000–250,000 cells were seeded the day before transfection. HiPSC-TLR were then transfected using Lenti-CGIP^12^, an eGFP-encoding plasmid of comparable size to PE-encoding pCMV-PE2^2^. In contrast to what was observed for the PEI method in 293-TLR cells, hiPSC transfection efficiency was strongly influenced by number of cells seeded the day before transfection (Fig. 3C). In the same experiment, cells were transfected side-by-side using pCMV-PE2 with g+4 R20P13_dP pegRNA. Measured PE efficiencies varied as well with the number of seeded cells, and optimum number of cells was comparable to what was observed for Lenti CGIP transfection (Fig. 3D). Regardless, full inter-experimental reproducibility was difficult to achieve and other PE efficiencies were measured for 50,000 and 100,000 cells seeded in other, temporally separated experiments (Supplementary Fig. 4E,F). Therefore, other factors aside of seeding cell number may influence transfection efficiency and/or prime editing efficiency itself in hiPSC, e.g. how many cells actually attach after seeding and what exact condition cells are in at the time of transfection.

### A selectable all-in-one CRISPR-PE piggyBac transposon allows for enrichment of desired point mutations in bulk cell populations

**Figure 4:**
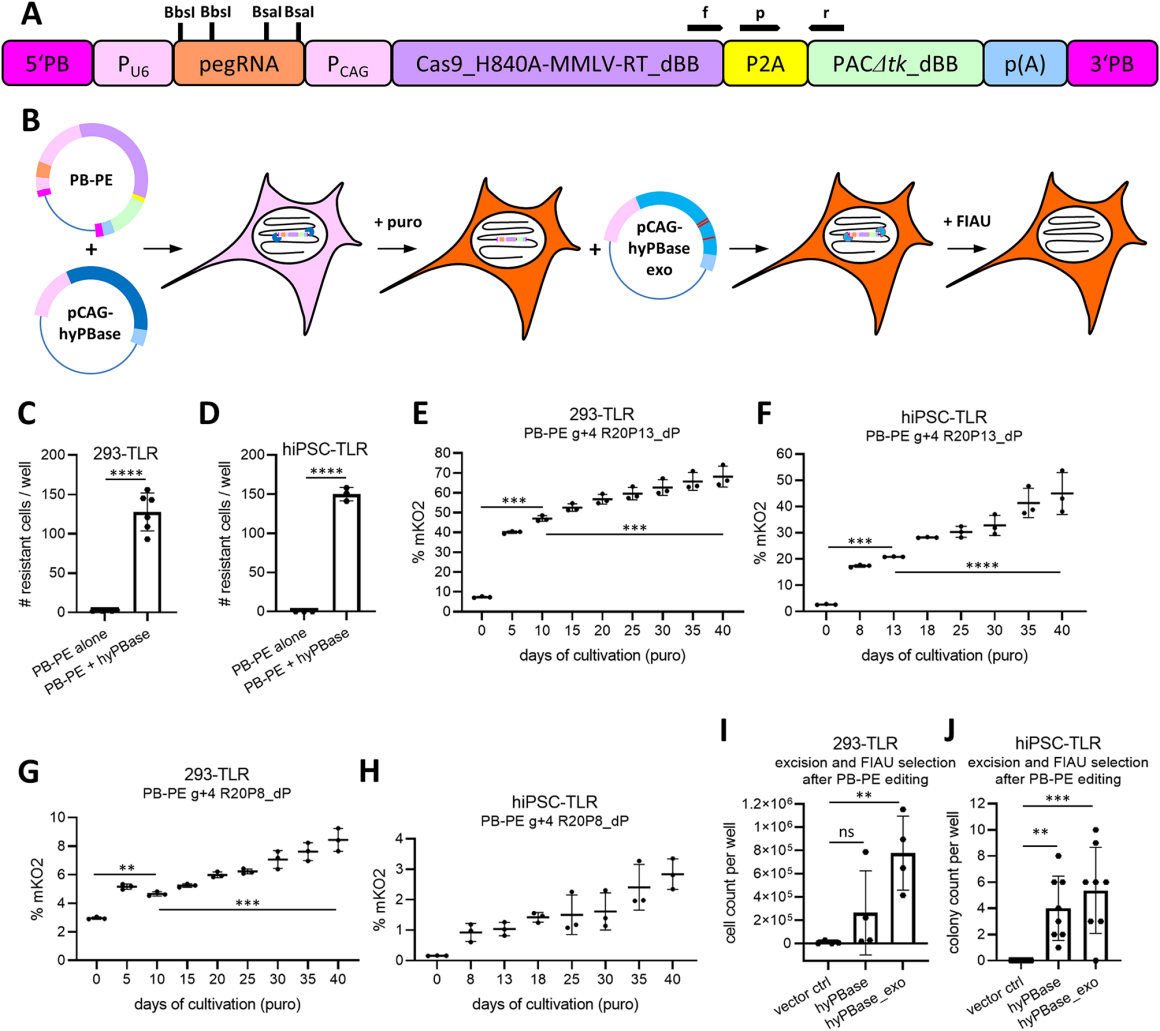
Proof-of-principle for a piggyBac prime editing (PB-PE) system allowing for enrichment of gene edited cells in human bulk cell populations. (**A**) Unscaled schematic of PB-PE vector. Cas9_H840A-MMLV-RT_dBB and PACΔtk_dBB are modified versions of PE2^2^ and puroΔtk^13^, respectively, where all Bbsl and Bsal sites were deleted. Approximate binding sites of PB-PE-specific qRT-PCR primers (f, r) and probe (p) are indicated as black tapered lines. (**B**) Graphic overview of a PB-PE based gene editing approach. (**C**, **D**) Calculated colony formation efficiency after puromycin selection of 293-TLR (**C**) and hiPSC-TLR (**D**) cells transfected with PB-PE transposon with and without hyPBase transposase. (**E**) FACS analysis for mKO2 gene corrected 293-TLR cells which were PEI-transfected with PB-PE g+4 R20P13_dP transposon and pCAG-hyPBase at different days of puromycin selection. (**F)** FACS analysis for mKO2 gene corrected hiPSC-TLR cells edited with PB-PE g+4 R20P13_dP transposon at different days of puromycin selection. (**G**, **H**) FACS analysis for mKO2 gene corrected 293-TLR (**G**) and hiPSC-TLR (**H**) cells edited with PB-PE g+4 R20P8_dP transposon at different days of puromycin selection. (**I**) Cell count per well of PB-PE g+4 R20P13_dP-edited 293-TLR cells, after transposon excision by hyPBase or hyPBase_exo (excision-optimized) transposase and FIAU counter-selection. (**J**) Colony count per well of PB-PE g+4 R20P13_dP-edited hiPSC-TLR cells, after transposon excision by hyPBase or hyPBase_exo transposase and FIAU counter-selection. Vector ctrl: cells transfected with L.CGIP (see Supplementary Methods). Data are represented as ± SD from n = 3 (**D**–**H**), n = 4 (**I**), n = 6 (**C**) or n = 8 (**J**) biological replicates and significance was calculated using 1-way ANOVA with Tukey's post-test except for (**C**) and (**D**) where unpaired t-test was used (****p ≤ 0.0001; ***p ≤ 0.001; **p ≤ 0.01; ns: p > 0.05).

In order to overcome limitations originating from suboptimal transfection methods or inter-experimental reproducibility, we sought to temporarily integrate prime editors into the genomes of cellular bulk populations and select for cells with successful integration. To this end, all-in-one CRISPR-PE cassettes were cloned into piggyBac (PB) transposon vectors. Therefore, a Cas9_H840A-MMLV-RT cassette based on the PE2 construct^2^ was joined via P2A to PAC*Δtk*, which allows for puromycin-based selection as well as fialuridine (FIAU)-based counter-selection after excision (Fig. 4A,B)^12,13^. Dual BbsI and BsaI sites were added as cloning sites for pegRNA protospacer and RTt-PBS and were removed from the rest of the vector sequence (dBB) in order to allow for fast and easy retargeting (Fig. 4A). 293-TLR cells transfected with both, PB-PE and hyperactive piggyBac transposase (hyPBase) vectors readily formed resistant colonies, whereas calculated colony formation efficiency was markedly reduced in cells transfected without transposase (Fig. 4C). For hiPSC-TLR similar results were found but no surviving colonies were detected in cells transfected without transposase (Fig. 4D; Supplementary Fig. 5A). In order to investigate whether extended PE expression after selection would be able to compensate for loss of edited cells when applying harsh transfection conditions, 293-TLR cells were PEI transfected with a PB-PE vector encompassing g+4 R20P13_dP pegRNA and pCAG-hyPBase and selected using puromycin. Flow cytometric analysis was performed every five days, at which points cells were also passaged. Increasing amounts of mKO2 positive cells were detected reaching almost 70% after extended cultivation time, whereas in transfected and unselected cells (corresponding to day zero) ~ 7% positive cells were measured (Fig. 4E). Transfection of hiPSC-TLR (150,000 cells seeded) with the same plasmids resulted in up to 50% of gene edited cells after extended cultivation time (Fig. 4F). In order to investigate whether extended PE expression using PB-PE vectors can also compensate for inefficient pegRNA designs, the previously evaluated low efficiency g+4 R20P8_dP pegRNA was cloned into the PB-PE vector. When overexpressed in 293-TLR, the mKO2 signal increased from 3% to 8.4% in the same time span of 40 days (Fig. 4G). Likewise, low efficiencies were found in hiPSC-TLR for this pegRNA design (Fig. 4H). Long-term expression of both pegRNA designs resulted in comparable and low overall eGFP signals in both, 293-TLR (0.123 ± 0.026%) and in hiPSC-TLR (0.024 ± 0.017%) (Supplementary Fig. 5B,C). An unguided control vector (PB-PE ugc) caused no change of eGFP or mKO2 signals over time (Supplementary Fig.5G).

The Cas9_H840A coding sequence of PB-PE was also modified to a version which allows targeting of PAMs consisting of NGAN and NGNG motifs (SpCas9-VQR)^14^. PB-PE VQR successfully targeted a GGTG motif on the bottom strand (VQR bot) and a CGAC motif on the top strand (VQR top) of the mKO2_mut sequence in 293-TLR, albeit at low efficiencies (Supplementary Figs. 5D and 7C,D). Similar to PB-PE g+4 R20P8_dP, prime editing with PB-PE VQR bot gradually and slowly increased mKO2 signal over time but overall underperformed when compared to efficient PB-PE g+4 R20P13_dP (Supplementary Fig. 5E). PB-PE was further modified to an xCas9 version, which was created to target NGT motifs^15^, while others have described that it was more efficient in targeting NGTC motifs^16^. PB-PE xCas9 with a ‘g0’ pegRNA successfully targeted a GGTC motif on the mKO2_mut sequence (detailed view in Supplementary Fig. 7E) in 293-TLR cells and increased the fraction of gene edited cells over time (Supplementary Fig. 5F). When tested side-by-side with other constructs, PB-PE xCas9 g0 construct outperformed PB-PE g+4 R20P8_dP, and it was ~ 25% as efficient as highly active PB-PE g+4 R20P13_dP (Supplementary Fig. 5F).

In order to analyze for potential TLR transgene silencing, 293-TLR cells expressing PB-PE g+4 R20P13_dP vector were cultivated side-by-side with and without hygromycin B for 5 days. No difference in the mKO2 signal was observed in any of the conditions, indicating that TLR was not silenced in 293-TLR cells (Supplementary Fig. 5H). Similar analysis in hiPSC-TLR revealed that ~ 20% of reporter activity was lost due to transgene silencing (Supplementary Fig. 5I). However, continuous cultivation of reporter cells under hygromycin selection pressure during genome editing would have affected eGFP-based read-out of indels in the current design of TLR, as cells with certain frame-shift indels would likely be ablated (Fig. 1A). Taking into account this limitation, actual gene correction efficiencies were overall slightly underestimated in hiPSC-TLR.

PB-PE gene-edited 293-TLR cells were furthermore analyzed by genomic PCR and Sanger sequencing. Cells edited with PB-PE g+4 R20P13_dP showed clear double peaks at the site of the G67D point mutation and the site where the PAM of g+4 was co-edited (Supplementary Fig. 6A). Faint additional chromatogram peaks were also detected in PB-PE g+4 R20P8_dP and in PB-PE VQR bot edited cells (Supplementary Fig. 6A). However and importantly, individual peak intensities in Sanger chromatograms are not strictly quantitative but depend on the exact analyzed sequence as well as applied reaction conditions. Thus, data from chromatograms corroborated FACS-based results only by trend, i.e. that PB-PE g+4 R20P13_dP was more efficient than PB-PE g+4 R20P8_dP and PB-BE VQR bot.

After mKO2 expressing cells had accumulated in the bulk populations by extended PB-PE g+4 R20P13_dP expression, 293-TLR and hiPSC-TLR cells were also transfected again using plasmid vectors encoding for hyPBase or an integrase-deficient variant^17^, termed hyPBase_exo (excision optimized) for excision of the PB-PE cassettes. Cells were then subjected to FIAU for thymidine kinase-based counter-selection. In 293-TLR cells treated with hyPBase, two out of four transfected wells had more surviving cells compared to vector control but results were not significant at the number of analyzed wells. In contrast, we counted substantially more surviving cells in all wells transfected with hyPBase_exo when compared to vector control, indicating that excised cells were successfully enriched (Fig. 4I). In PB-PE edited hiPSC-TLR, transfection of expression vectors for both, hyPBase and hyPBase_exo, resulted in comparable surviving colony numbers after FIAU selection, whereas no surviving colonies were found in vector control transfected cells (Fig. 4J). Specific quantitative real time qPCR primer/probe-sets were designed for detection of PB-PE transposons, matching in the region of the P2A joint spanning from the MMLV-RT to the PAC sequence (Fig. 4A, Supplementary Table 4). Genomic qPCR showed that detection of PB-PE transposons was strongly decreased, albeit in not fully absent, in hyPBase_exo excised and FIAU selected 293-TLR and hiPSC-TLR (Supplementary Figs. 6C,D).

### A practical approach employing PB-PE for genetic correction of an ALS-related point mutation

**Figure 5:**
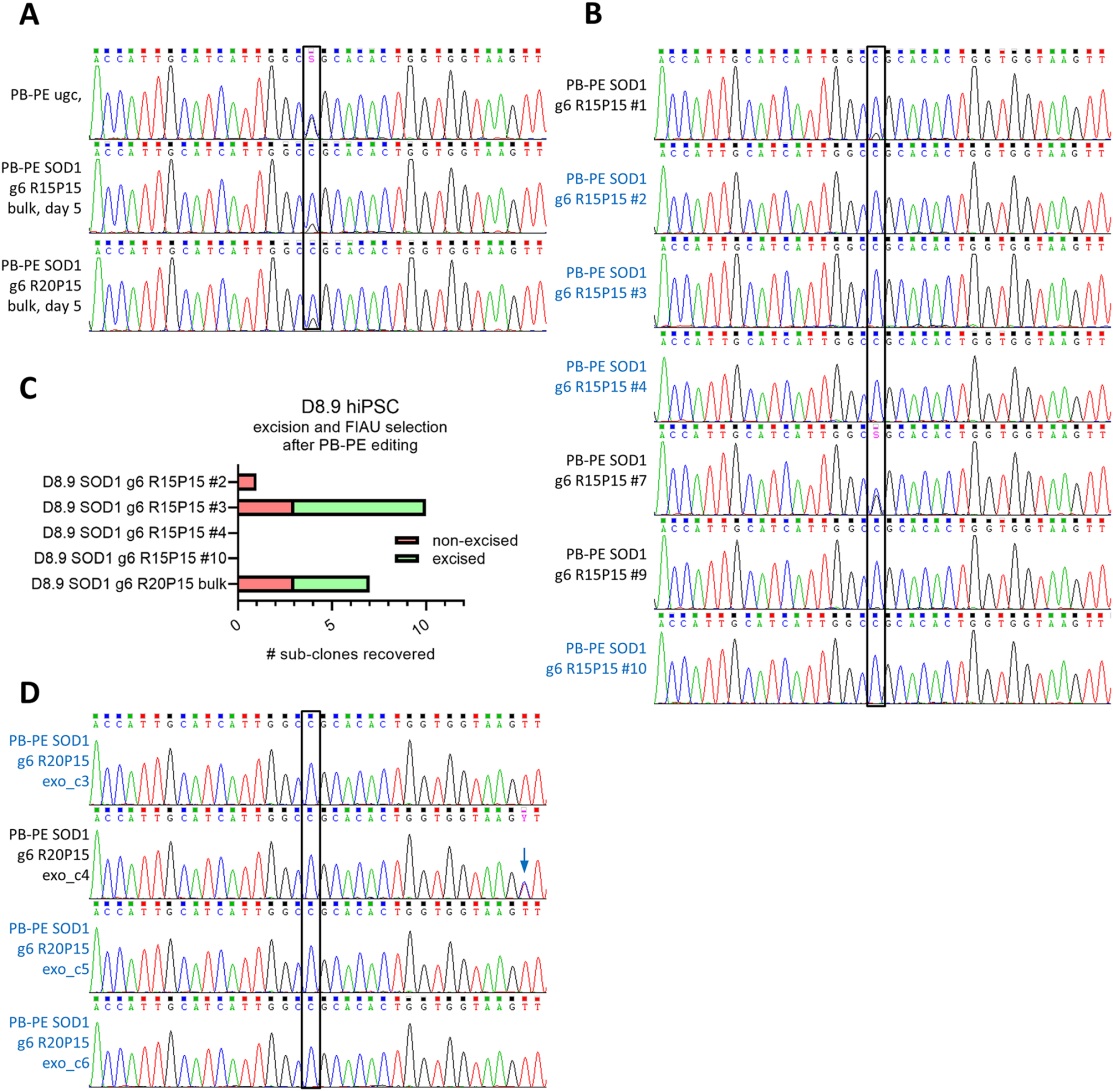
PB-PE gene editing of SOD1_R115G (**A**) Sanger chromatograms of D8.9 hiPSC harboring a mono-allelic SOD1_R115G point mutation after transfection of PB-PE vectors and 5 days of puromycin selection. Two different vectors were employed, one with a 15 nt RTt (PB-PE SOD1 g6 R15P15) and one with a 20 nt RTt (PB-PE SOD1 g6 R20P15). Unguided PB-PE vector served as control (PB-PE ugc). (**B**) Chromatograms of seven sub-clones directly established from PB-PE SOD1 g6 R15P15 treated cells. Fully gene corrected clones are denoted in blue font. (**C**) Colony counts of PB-PE edited D8.9 hiPSC clones and a gene edited bulk population after hyPBase_exo transfection and FIAU selection. Excised clones as determined by PB-PE qRT-PCR are shown in light green, non-excised clones are shown in light red. (**D**) Chromatograms of the four excised and PB-PE edited sub-clones established from a PB-PE SOD1 g6 R20P15 treated bulk population. Sub-clones with fully correct SOD1 sequences are denoted in blue font. Blue arrow: additional mutation, likely introduced by 'read-through' into pegRNA scaffold.

In order to assess PB-PE in a more hands-on application, the construct was adapted to target and correct a R115G (C>G) point mutation in the SOD1 gene. SOD1_R115G has been identified as a gain-of-function mutation causing familial amyotrophic lateral sclerosis (ALS)^4^. D8.9 hiPSC derived from a patient with ALS harboring a mono-allelic SOD1_R115G mutation were transfected with pCAG-hyPBase and PB-PE SOD1 g6 R15P15 or PB-PE SOD1 g6 R20P15. Already after 5 days of puromycin selection, distinct shifts were visible at the site of the R115G point mutation in Sanger chromatograms (Fig. 5A). From PB-PE SOD1 g6 R15P15 treated cells individual clones were directly picked and expanded. Thereof, three clones (#1, #7, #9) were not yet fully edited and (minor) double peaks were detectable (Fig. 5B). However, four clones (#2, #3, #4, #10) no longer showed a visible double peak in the sequenced PCR products, indicating a high degree of correction (Fig. 5B). PB-PE SOD1 g6 R20P15 treated cells were cultivated for extended time in order to allow for more cells to be edited (Supplementary Fig. 6B). Thereafter, PB-PE SOD1 g6 R15P15 treated clones #2, #3, #4 and #10 as well as the PB-PE SOD1 g6 R20P15 treated bulk population were transfected using pCAG-hyPBase_exo and subjected to FIAU for counter-selection. Surprisingly, no surviving colonies were observed after selection of PB-PE SOD1 g6 R15P15 clones #4 and #10, whereas one resistant colony was found for clone #2 and ten colonies were found for clone #3 (Fig. 5C). Genomic qPCR analysis revealed a stronger detection of PB-PE in PB-PE SOD1 g6 R15P15 clones #4 and #10, suggesting that multiple copies may be integrated in these clones. Likewise, a rather low PB-PE qPCR signal was detected in PB-PE SOD1 g6 R15P15 #3, suggesting a relatively lower copy number. PB-PE qPCR also revealed that the FIAU-selected sub-clone PB-PE SOD1 g6 R15P15 #2 was not excised, while seven out of the ten sub-clones originating from PB-PE SOD1 g6 R15P15 #3 were indeed excised (Fig. 5C).

In order to reduce clonal bias for successful transposon excision, PB-PE SOD1 g6 R20P15 treated bulk population was transfected with hyPBase and FIAU-selected. Seven surviving colonies were found, out of which four were excised (Fig. 5C). Sanger analysis revealed that those four clones were fully gene corrected, albeit the clone PB-PE SOD1 g6 R20P15_exo_c4 showed incorporation of an additional base which likely originated from the pegRNA expression scaffold (Fig. 5D, Supplementary Fig. 7F). Together, an experimental approach of excising PB-PE from a gene edited bulk population was more reliable than excision from single cell clones. It is possible that both, total numbers and specific genomic integration sites of PB-PE vectors may affect how efficiently they can be excised again. In a bulk population such unpredictable parameters can be better compensated for and thus this may be a more hands-on protocol for cases where the PB-PE transgene should be removed after editing.

## Discussion

CRISPR-PEs are novel and versatile tools for introducing distinct modifications into the genome but their practical application can be hampered by transfection efficiency and pegRNA design. This is of particular importance when considering applications in bulk populations and at larger scales. Here, we developed a system which allows us to analyze efficiencies of different CRISPR-PE designs and plasmid transfection protocols, employing a lentiviral vector-based TLR system displaying indel formation with a frame-shifted eGFP and gene correction by activation of an mKO2_G67D mutant gene. At first, we also employed an ABE vector for mKO2 gene correction, which resulted in low concurrent indel formation. However, correction efficiencies for the analyzed reporter sequence were several-fold lower than previously reported for ABEs^1,2^. The ABE7.10 version which we used here was reported to efficiently mediate A → G conversion of 17–14 nt PAM-distally located adenines^1^. We had purposely introduced a PAM for the g+17 gRNA in our TLR vector in order for the adenine of the G67D point mutation to be located 15 nt PAM-distally. While further investigation of the issue of low ABE efficiency at our specific target sequence is needed, we noticed that indel formation with a Cas9_D10A nickase was several-fold lower when using g+17 gRNA (0.070 ± 0.003%) compared to g+1 gRNA (0.411 ± 0.073%) (Supplementary Fig. 2I). For efficient ABE-based adenine conversion an initial Cas9_D10A nickase step has to occur which then allows for adenosine to inosine conversion by the engineered TadA enzyme^1^. Furthermore, our designed mKO2 sequence contains another adenine which is 12 nt PAM-distally located. It was previously reported that A- > G conversion can also occur at 12 nt PAM-distal adenines, with 10–15% relative efficiency compared to 15 nt PAM-distal adenines^1^. A → G conversion at this location would lead to H68R missense mutation and we found that mKO2 was indeed not fluorescent when introducing H68R. Therefore, we suspect that some of the mKO2 signal may have been lost due to additional editing at this position.

In contrast to ABEs, CRISPR-PEs are generally able to concurrently introduce a PAM disruption together with the intended modification and it has been shown that this technique improved introduction of desired modifications and reduced indel formation^2^. In our reporter system we found that concurrent PAM mutation improved mKO2 gene correction and reduced indel formation as well. We also observed that (undesired) indel introduction by CRISPR-PE was more related to the exact targeted sequence rather than the RTt and PBS of a given pegRNA. All g+4 pegRNAs, which target a sequence just three nucleotides downstream of g+1 pegRNAs resulted in several-fold lower indel formation even without concurrent introduction of a PAM mutation. Interestingly, this was not reflected in a Cas9 nuclease-based knockout experiment, where transfection of g+4 sgRNA resulted in higher indel formation than g+1 sgRNA. However, the g+4 sgRNA likely displays some bias in our TLR-system, as because its target has a ‘T’ at the PAM -4 position, which frequently results in + 1 nt insertions^10^, thereby shifting the eGFP(+ 2) gene into the active frame. Indeed, we found that a higher fraction of analyzed eGFP activating disruptions were + 1 nt insertions when using Cas9 nuclease with g+4 than compared to g+1 (Supplementary Fig. 3). Nevertheless, the mechanism by which nickase constructs such as Cas9_H840A introduce indels may be distinct from nuclease-based indel formation^18^. Moreover, our data suggests that at least some cases of indel formation by CRISPR-PE may be explained by improper flap resolution, while others may have resulted from re-cleavage of already edited sequences (Supplementary Fig. 3). Together, our data shows that rates of PE-based indel formation is difficult to predict by simple Cas9 nuclease experiments and other methods should be considered for definition of good protospacers in order to avoid high indel rates at a target locus of interest. Overall, proper pegRNA design comprises of high activity and low indel rate. Finding a faithful pegRNA for the purpose of installing a certain mutation is generally driven by how many clones with desired sequences versus how many clones with undesired sequences can be isolated. On the side of undesired sequences both, sequences with indels as well as non-edited sequences should be counted. In other words, if a certain pegRNA is highly active in installing the desired mutation but at the same time displays a very high indel rate, we deem that this would be a rather improper design. This is especially true for cases where PAM disruption cannot be applied. If the indel rate of a pegRNA is found to be rather low (i.e., as we observed for all g+4 pegRNAs in the TLR target), its activity should be optimized by variation of RTt and PBS lengths.

While finding an optimized pegRNA design for efficient and faithful introduction of a desired modification at a given target locus remains an important focus, we also noticed that efficiency of CRISPR-PE gene correction strongly depends of availability of good and reproducible transfection protocols. Although we were able to efficiently transfect both, HEK293 cells as well as hiPSCs, a considerable fraction of CRISPR-PE edited HEK293 cells were lost some days after transfection. We observed this effect with several transfection agents, but the relative loss was especially high when using the PEI transfection protocol. Thus, we suspect that toxicity of transfection agents may play a role in the loss of gene edited cells. Contrarily, we did not observe loss of gene edited cells after transfection in hiPSCs. However, we noticed that PE efficiency was strongly dependent on the amount of cells seeded before transfection and we show that there is reason to suspect that additional, yet unknown factors may play a role as well. Allover, highly reproducible protocols for efficient prime editing were difficult to establish in hiPSCs. In order to address those issues, we explored the possibility for extended expression of PEs. Our data shows that PB-PE, a combination of selectable all-in-one PE cassettes with piggyBac transposon technology allows for enrichment of desired genetic modifications in 50% or more in bulk cell populations, even when initial transfection conditions were not optimal. Importantly, forced expression of ‘all-in-one’ PB-PE cassettes leads to a fast and profound increase of modified cells when using a previously optimized pegRNA. Employment of less efficient pegRNA designs resulted in a steady but very slow increase of gene edited cells and no strong boost of gene correction was observed after 40 days of continuous expression. While fully deciphering this somewhat surprising finding will require further investigation, our data clearly demonstrates that inadequate design of pegRNAs cannot be easily overcome by extended PE expression. Thus, proper evaluation of pegRNAs is fundamental for the general outcome of PE-based genome editing and in the meantime, algorithm-based design for promising pegRNA designs is possible^3^. Interestingly, in PB-PE g+4 R20P8_dP and PB-PE VQR bot-transfected cells, more mKO2 positive cells were detected at day 5 of cultivation than at day 10 (Fig. 4G, Supplementary Fig. 5E). Possibly, at day 5 some transiently transfected cells without integration of PB-PE constructs into the genome were still present. Such cells would contribute to temporarily increased reporter activity but would later be lost again during extended puromycin selection. In case of highly efficient g+4 R20P13_dP pegRNA such an effect could be masked due to high overall reporter activity already seen on day 5. Strikingly, the VQR bot pegRNA was designed in an almost identical manner as the highly efficient g+4 R20P13_dP pegRNA (Supplementary Fig. 7B,D). However, in a long-term expression experiment PB-PE VQR bot was rather underperforming. This may in part be attributable to lower recruitment efficiency of Cas9 VQR to NGTG PAMs^19^. A full investigation of this issue would require a new TLR design which contains a stronger canonical PAM for Cas9 VQR, such as NGAG. Nevertheless, we think that further expansion of the PAM repertoire of PB-PE vectors to PAMs such as NGN, NRN and NYN (Cas9-SpG and Cas9-SpRY)^16^ or NAAN (Cas9-iSpyMac)^20^ should be possible.

Overall, we envision PB-PE vectors for the fast and efficient examination of potential usefulness of different pegRNA designs in target cell types of choice. An efficient pegRNA ought to introduce easily detectable changes in a chromatogram within short time of cultivation after introduction and selection of PB-PE vectors, whereas absence of such an observation would likely indicate a design which needs further improvement. While other methods are out there for quick estimation of pegRNA efficiency, such as in silico predictions^3^, the PB-PE method followed by Sanger sequencing analysis is a fast and simple hands-on approach which will provide more accurate and direct experimental answers at a defined target locus in a designated cell line. In cases where a highly reliable transfection technique for a certain cell line is established, genomic integration of prime editors may not be required for estimation of pegRNA efficiency. However, in case of hiPSC-TLR we observed that the generally efficient g+4 R20P13_dP pegRNA activated only 2.5% of the mKO2 signal without selection (Fig. 4F), most likely due to ineffective transfection. After only 8 days of selection, this value increased to 17.4%, which would be detectable by simple Sanger sequencing^21^. Meanwhile, changes introduced by an inefficient pegRNA would be difficult to detect at this point. Thus, enrichment of PE-expressing cells by methods such as the presented PB-PE system can aid in fast evaluation of pegRNA efficiency by Sanger sequencing without the need for highly effective transfection protocols.

PB-PE is also equipped with a thymidine kinase for FIAU- or gancyclovir-based counter-selection if/when excision of the transposon is desired for more sophisticated applications and we demonstrate that especially employment of an integrase-deficient transposase^17^ followed by FIAU selection efficiently enriches counter-selected cells. Based on the studies presented here, we do recommend a strategy of direct excision of PB-PE transgenes from a gene edited bulk population (followed by sub-cloning of FIAU-selected, surviving hiPSC), rather than generation of gene edited sub-clones followed by excision, as some sub-clones may be ‘hard-to-excise’. Also we highly recommend employing additional techniques such as the presented PB-PE qPCR and/or Southern blot for confirmation of transposon excision. Success of FIAU selection is dependent on expression of the truncated HSV thymidine kinase present in PB-PE vectors. Transgene silencing can lead to escape from FIAU selection, especially in cell lines which are more prone for such processes, such as pluripotent stem cells. For the same reason, we recommend to cultivate cells with integrated PB-PE transposons in puromycin containing medium until time point of transfection with integrase-deficient hyPBase_exo vector.

Finally, we have successfully applied PB-PE for gene correction of the SOD1_R115G mutation in ALS-patient-derived hiPSC. Disease modeling employing parental cells and syngenic gene corrected cells will help to better understand the biological mechanism associated with this rare hereditary mutation^22^.

## Methods

### Cell lines

HEK293T cells were a kind gift from Dr. Axel Schambach at Hannover Medical School, Hannover, Germany and HEK293 cells were obtained from DMSZ Braunschweig, Germany (ACC 305). They were cultivated in DMEM 4.5 g/L glucose GlutaMAX (Gibco, Thermo Fisher Scientific #31966021) with 100 × Pen/Strep and 10% FBS. Generation and maintenance of transgene-free phoenix hiPSC, also known as hHSC_Iso4_ADCF_SeV-iPS (https://hpscreg.eu/cell-line/MHHi001-A), was described elsewhere^11^. Briefly, phoenix hiPSC were maintained in ‘homemade’ Essential 8 (E8) medium and cultivated on MatriGel hESC-qualified matrix (Corning #354277), according to manufacturer’s instructions. For passaging, hiPSC were detached every 5–6 days using Accutase solution (Sigma-Aldrich, Merck #A6964) and 50,000 cells were seeded per well of 6-well in E8 medium with 10 μM Y-27632 ROCK inhibitor (produced at the Institute of Organic and Technical Chemistry, Leibniz University Hannover). Monoallelic SOD1_R115G hiPSC (also known as D8.9) were derived from a 59 year old male patient suffering from autosomal-dominant familial ALS (brother and mother affected). They were generated with ethical permits EK45022009/EK39312201 and characterization of the iPSC line was recently reported^22^. D8.9 hiPSC were adapted to E8/MatriGel conditions prior to gene correction using PB-PE constructs and from thereon out cultivated as described for phoenix hiPSC. All methods involving the use of human cell lines were carried out in accordance with institutional guidelines of Hannover Medical School and were approved by the authorities.

### Lentiviral vector preparation and generation of TLR bulk cell populations

Lentiviral vectors were produced by transient transfection of Lenti-TAp (encoding a TLR vector with a PAC gene) or Lenti-TAh (encoding a TLR vector with a HPH gene) plasmids along with pCDNA3.GP.4xC, pMD2.G and pRSV-Rev into HEK293T cells using the CaCl2 protocol as described previously^12^. Vectors were titrated by transduction of serial-fold dilutions of 1:100–1:300,000 on HEK293T cells and performance of qPCR on isolated genomic DNA (gDNA) using primer/probe pairs for the WPRE element encoded on lentiviral vectors and PTBP2 for internal reference^23^. For generation of 293-TLR reporter cells, 600,000 cells of HEK293 were transduced in culture medium supplemented with 8 μg/ml protamine sulfate (Sigma-Aldrich, Merck #P3369) with Lenti-TAp or Lenti-TAh vector particles at MOI = 0.02 and selected using 3 μg/ml puromycin (Sigma-Aldrich, Merck #P8833) in case of Lenti-TAp or 500 μg/mL hygromycin B (Gibco, Thermo Fisher Scientific #10687010) in case of Lenti-TAh. The calculated clonality was ~ 12,000 different reporter cell clones for each reporter bulk population. Similarly, HEK293 were also transduced with Lenti-TAp at MOIs of 0.1, 0.5, 1, 2 and 5 and selected using puromycin. Phoenix hiPSC reporter cells were generated by transduction of 600,000 cells using Lenti-TAh. In lab internal protocols, transduceability of hiPSC by VSV-G-coated lentiviral vectors was around eight-fold lower compared to HEK cells and thus a calculated MOI of 0.16 was applied. Transduced phoenix iPSC were selected using 100 μg/mL hygromycin B. All reporter cell bulk populations were enriched for the eGFP-mKO2- fraction by FACS sorting prior to their employment in reporter assays. This was necessary to omit rare events in which mKO2 or eGFP was activated by random mutations introduced during the reverse transcription step of lentiviral vector production. At least 250,000 cells of each reporter bulk population were kept in culture at all times in order to maintain clonal diversity.

### Transfection methods

For all assays with 293-TLR cells, 80,000 cells per well of 12-well were seeded the day before transfection, unless stated otherwise. For polyethlyenimine (PEI; Sigma-Aldrich, Merck #408727) transfections, cells were transfected with 0.5 μg of plasmid encoding sgRNA or pegRNA and 0.5 μg of CRISPR plasmid per well: pU6-(BbsI)_CBh-Cas9-T2A-BFP^6^ in case of nuclease assays, Cas9_D10A-BFP in case of nickase assays, pCMV-ABE7.10^1^ or pCBh-ABE7.10-BFP for adenine base editing, and pCMV-PE2^2^ for prime editing. For assays including ssODNs, the amounts of sgRNA and CRISPR plasmids were reduced to 0.375 μg each and ssODNs were added at 14 nM final concentration in the ready transfection solution, unless indicated otherwise. Briefly, plasmids and ssODNs were diluted in 48.75 μL DMEM 4.5 g/L glucose GlutaMAX and 1.25 μL 0.1 mM PEI was added and incubated 10 min at room temperature before adding 300 μL DMEM 4.5 g/L glucose GlutaMAX with 10% FBS. Medium was fully aspirated from 293-TLR cells and 350 μL of ready transfection solution was added per well. Cells were then incubated for 4 h at 37 °C and transfection solution was aspirated and replaced with 3 mL regular cultivation medium. For Lipofectamine LTX (Thermo Fisher Scientific #15338100), TransIT-LT1 (Mirus Bio #2300) and FuGENE 6 (Promega #E2691) transfections, each manufacturer’s specific instructions were followed. Cells were transfected with same total and relative amounts of plasmids per well as described for PEI. The following transfection agent volumes per well were tested: for Lipofectamine LTX 1.5 μL and 3.0 μL, for TransIT-LT1 2.2 μL and 4.4 μL and for FuGENE 6 1.5 μL and 3.0 μL. For all three low toxicity agents, transfections with a higher volume of agent were superior and protocols were accordingly set up for TLR assays.

For standard transient transfection assays with phoenix hiPSC-TLR cells, 150,000 cells were seeded in E8 medium with 10 μM Y27632 Rho Kinase inhibitor per well of MatriGel-coated 12-well plate one day before transfection, unless stated otherwise. Cells were transfected using Lipofectamine™ Stem Transfection Reagent (LFS; Thermo Fisher Scientific #STEM00015) with 0.34 μg of plasmid encoding sgRNA or pegRNA and 0.34 μg of CRISPR plasmid per well. Briefly, plasmids were diluted in 50 μL OptiMEM (Gibco, Thermo Fisher Scientific #31985070) supplemented with 40 ng/mL human bFGF (OM + bFGF) and mixed 1:1 with 2 μL LFS diluted in 48 μL OM + bFGF and incubated for 10 min at room temperature. Culture medium was fully aspirated and replaced with 200 μL OM + bFGF and 100 μL transfection solution was added on top. Transfection was incubated for 4 h at 37 °C before adding 2 mL of E8 medium without Pen/Strep. TLR assays were analyzed by flow cytometry 120 h post transfection, unless indicated otherwise.

### PB-PE assays and SOD1_R115G gene editing

For PB-PE assays in 293-TLR, cells were PEI-transfected with 0.95 μg of respective PB-PE construct and 0.05 μg of pCAG-hyPBase. Cells were split and FACS analyzed 96 h post transfection for obtaining the measurement corresponding to zero days after selection and 3 μg/mL puromycin was added from thereon out. Cells were then split onto new 12-well plates at each time point of FACS analysis and eGFP and mKO2 expression was analyzed.

For PB-PE assays in phoenix hiPSC-TLR, 150′000 cells were seeded the day before transfection. Cells were transfected with 0.95 μg of respective PB-PE construct and 0.05 μg pCAG-hyPBase using the above LFS protocol. Cells were split and FACS analyzed 72 h post transfection for obtaining the measurement corresponding to zero days after selection and 1 μg/mL puromycin was added from thereon out. Cells were then split onto new 12-well plates at each time point of FACS analysis and eGFP and mKO2 expression was analyzed.

For editing of SOD1_R115G point mutation, D8.9 hiPSC were transfected with 0.95 μg of respective PB-PE construct and 0.05 μg pCAG-hyPBase employing the above LFS protocol for hiPSC-TLR and except that 1.75 μL LFS was used per well and 165,000 cells were seeded per well the day before transfection. Cells were selected using 1 μg/mL puromycin from day 3 post transfection.

### PB-PE excision and thymidine kinase-based counter-selection

PB-PE g+4 R20P13_dP edited 293-TLR cells, which had been selected with puromycin and grown for 40 days, were seeded on a 12-well plate for PEI transfection as described above. Importantly, cells were cultivated in medium with 3 μg/mL puromycin until time-point of transfection, in order to reduce potential silencing of the PB-PE cassette, which co-expresses HSV-thymidine kinase (*Δtk*). Cells were transfected using 1 μg Lenti CGIP as vector control or pCAG-hyPBase or pCAG-hyPBase_exo and cultivated for 4 days in medium without selective agents. 293-TLR cells were then split 1:10 onto new wells of 12-well and selected using 5 μM fialuridine (FIAU; Sigma-Aldrich #SML0632) from day 5 to 19 post transfection, at which point surviving cells were counted for each well.

PB-PE g+4 R20P13_dP edited hiPSC-TLR as well as PB-PE SOD1 g6 R15P15 and PB-PE SOD1 g6 R20P15 edited D8.9 hiPSC were cultivated in medium with 1 μg/mL puromycin until time-point of transfection. Cells were transfected using 1 μg Lenti CGIP as vector control or pCAG-hyPBase or pCAG-hyPBase_exo employing the above LFS-based protocol and were subjected to 1 μM FIAU starting from day 5 post transfection. 5 days of FIAU selection was sufficient for ablation of non-excised hiPSCs.

### PB-PE qPCR

Genomic gDNA from PB-PE edited and thereof excised 293-TLR, hiPSC-TLR and D8.9 hiPSC was isolated using GenElute™ Mammalian Genomic DNA Miniprep Kit (Merck, Sigma-Aldrich #G1N350). A primer/probe mix was set up for each target (PB-PE and PTBP2), containing 3.3 µM final concentration of each, forward and reverse primer and 1.5 µM of respective probe (for sequences see Supplementary Table 4). qPCR assays were set up per well of 96-well plate as follows: 7.5 µl TaqMan™ Gene Expression Master Mix (Thermo Fisher # 4369016) + 1.5 µL primer/probe + 20–100 ng of gDNA sample in 6 µL ddH_2_O. Standard curves for both targets were set up as ten-fold dilution steps containing 1,000,000–1000 copies of PB-PE or pqPCR-Stdx4 plasmids. The latter contains the target sequence for PTPB2^23^.

### Genomic PCRs and TOPO cloning

Genomic PCRs for analysis of mKO2_G67D and SOD1_R115G point mutations were performed using Phusion™ Hot Start II High-Fidelity DNA-Polymerase (Thermo Fisher Scientific #F549L) in HF buffer with 2 ng/μL of respective genomic DNA template, 0.2 mM of each, dATP, dGTP, dCTP and dTTP and 0.2 μM of each primer (mKO2 PCRs: mKO2 gen for + mKO2 gen rev; SOD1 PCRs: SOD1 for #2 + SOD1 rev #2; Primer sequences are listed in Supplementary Table 1). PCR program was set according to manufacturer’s instructions using 64 °C annealing for mKO2 PCRs and 60 °C for SOD1 PCRs with 20 s extension time for mKO2 PCRs and 15 s for SOD1 PCRs, for a total of 35 cycles. For detailed analysis of indels from Cas9 nuclease assays and PE assays (Supplementary Fig. 3), mKO2 PCR products were sub-cloned using TOPO™ TA Cloning™ Kit for Sequencing (Thermo Fisher Scientific #450030) according to manufacturer’s instructions and analyzed by Sanger sequencing.

### Plasmids and Oligos

A detailed description of all molecular cloning procedures for plasmids first used in this publication, as well as primer sequences and ssODN sequences are provided in Supplementary Methods. Plasmids pU6-(BbsI)_CBh-Cas9-T2A-BFP^6^, pCMV-ABE7.10^1^, pCMV-PE2^2^ and lenti-Cas9-VQR-Blast^24^ were obtained via Addgene (#64323; #102919; #132775; #87155). The pCMV-hyPBase^13^ plasmid was obtained via Wellcome Trust Sanger institute and the mKO2-hCdt1(30/120)/pCSII-EF (FUCCI-Red)^25^ plasmid was kindly provided by Atsushi Miyawaki. Plasmids and cell lines generated for this study and can be requested from the corresponding authors. PB-PE and PB-PE VQR plasmids have been deposited at Addgene (#162796; #162797). PB-PE xCas9 will be available from Addgene in the same folder. Importantly, it is strongly recommend to use a bacterial strain with reduced recombination activity suitable for amplification of repetitive elements for sub-cloning of all PB-PE constructs, such as NEB Stable Competent E. coli (NEB #C3040H). Also, PB-PE plasmids should be grown at 30 °C instead of 37 °C at all steps, as their rather large size (~ 13 kb) makes them prone to recombination. The plasmids pCAG-hyPBase and pCAG-hyPBase_exo can be requested from the authors if an MTA was signed with Wellcome Trust Sanger for the pCMV-hyPBase plasmid beforehand. Alternatively, they can be generated using the detailed molecular cloning strategy as described in Supplementary Methods.

### Statistical methods

All error bars represent positive and negative standard deviation calculated from at least n = 3 independent measurements. An independent measurement was classified as a spatially separated transfection. Statistical evaluation of data presented in columns was performed using one-way ANOVA with Tukey’s post-test at 95% CI, whereas data shown in groups was analyzed using two-way ANOVA and Bonferroni multiple comparisons test. Data presented as two single groups was analyzed by two-tailed unpaired t-test.

## Supplementary Information

Supplementary Information 1.

Supplementary Information 2.

## Data Availability

All raw data sets generated during this study and presented here are available from the corresponding authors on request.
