# Supplementary material for: A selectable all-in-one CRISPR prime editing piggyBac transposon allows for highly efficient gene editing in human cell lines
Source: Sci Rep. 2021 Nov 12;11:22154. doi: 10.1038/s41598-021-01689-2 (PMC8589839; doi:10.1038/s41598-021-01689-2)
Supplement: Supplementary file 1 — Supplementary Information 1. [file 41598_2021_1689_MOESM1_ESM.pdf]

## Supplementary Figures:

# A selectable all-in-one CRISPR prime editing piggyBac transposon allows for highly efficient gene editing in human cell lines

Reto Eggenschwiler[1,2]*, Thomas Gschwendtberger[3,4], Christian Felski[1,2], Christopher Jahn[1,2], Florian Langer[1,2], Jared Sterneckert[5], Andreas Hermann[6,7], Jonathan Lühmann[8], Doris Steinemann[8], Alexandra Haase[2,9,10], Ulrich Martin[2,9,10], Susanne Petri[3,4], Tobias Cantz[1,2,11]*

[1]Research Group Translational Hepatology and Stem Cell Biology, Department of Gastroenterology, Hepatology and Endocrinology, Hannover Medical School, 30625 Hannover, Germany.

[2]REBIRTH-Research Center for Translational Regenerative Medicine, Hannover Medical School, 30625 Hannover, Germany.

[3]Department of Neurology, Hannover Medical School, 30625 Hannover, Germany.

[4]Center for Systems Neuroscience, Hannover Medical School, 30625 Hannover, Germany.

[5]Technische Universität Dresden, Center for Regenerative Therapies Dresden (CRTD), 01307 Dresden, Germany

[6]Translational Neurodegeneration Section „Albrecht-Kossel", Department of Neurology and Center for Transdisciplinary Neurosciences Rostock (CTNR), University Medical Center Rostock, University of Rostock, Rostock, Germany

[7]German Center for Neurodegenerative Diseases (DZNE) Rostock/Greifswald, 18147 Rostock, Germany

[8]Institute of Human Genetics, Hannover Medical School, 30625 Hannover, Germany

[9]Leibniz Research Laboratories for Biotechnology and Artificial Organs (LEBAO), Department of Cardiothoracic, Transplantation and Vascular Surgery, Hannover Medical School, 30625 Hannover, Germany.

[10]Biomedical Research in Endstage and Obstructive Lung Disease (BREATH), German Center for Lung Research (DZL), 30625 Hannover, Germany.

[11]Max Planck Institute for Molecular Biomedicine, Cell and Developmental Biology, 48149 Münster, Germany.

*correspondence: R.E.: eggenschwiler.reto@mh-hannover.de; T.C.: cantz.tobias@mh-hannover.de

# Supplementary Figure 1

**A**

| AA pos. (mKO2) | 57 | 58 | 59 | 60 | 61 | 62 | 63 | 64 | 65 | 66 | 67 | 68 | 69 | 70 | 71 | 72 | 73 | 74 | |
|---|---|---|---|---|---|---|---|---|---|---|---|---|---|---|---|---|---|---|---|
| mKO2 | F | D | L | V | S | H | V | F | C | Y | G | H | R | V | F | T | K | Y | |
| mKG | F | D | L | V | S | H | V | F | A | Y | G | H | R | V | F | T | K | Y | |
| GFP1 | F | D | I | L | T | A | A | F | C | Y | G | N | R | C | F | V | N | Y | |
| GFP-like YFP | F | D | I | L | T | A | T | F | C | Y | G | N | R | C | F | C | E | Y | |
| GFP-like RFP | F | D | L | L | S | H | T | F | C | Y | G | N | R | P | F | T | K | Y | |
| GFP-like GFP | F | D | I | L | S | A | A | F | C | Y | G | N | R | C | F | T | K | Y | |
| GFP | F | D | I | L | S | A | T | F | T | Y | G | N | R | C | F | C | D | Y | |
| Amcyan | F | D | I | L | S | T | V | F | M | Y | G | N | R | C | F | T | A | Y | |
| DsRedEx | W | D | I | L | S | P | Q | F | Q | Y | G | S | K | V | Y | V | K | H | |
| dTom | W | D | I | L | S | P | Q | F | M | Y | G | S | K | A | Y | V | K | H | |
| E2-Crimson | W | D | I | L | S | P | Q | F | F | Y | G | S | K | A | Y | I | K | H | |
| mCherry | W | D | I | L | S | P | Q | F | M | Y | G | S | K | A | Y | V | K | H | |
| mOrange | W | D | I | L | S | P | Q | F | T | Y | G | S | K | A | Y | V | K | H | |
| eYFP | W | P | T | L | V | T | T | F | G | Y | G | L | Q | C | F | A | R | Y | |
| mVenus | W | P | T | L | V | T | T | L | G | Y | G | L | Q | C | F | A | R | Y | |
| eGFP | W | P | T | L | V | T | T | L | T | Y | G | V | Q | C | F | S | R | Y | |
| eBFP2 | W | P | T | L | V | T | T | L | S | H | G | V | Q | C | F | A | R | Y | |
| mCerulean | W | P | T | L | V | T | T | L | T | W | G | V | Q | C | F | A | R | Y | |
| mKO2 A2G_Hc | F | D | L | V | S | H | V | F | C | Y | G | R | R | V | F | T | R | Y | not fluorescent |
| mKO2 A2G_2_Hc | F | D | L | V | S | H | V | F | C | Y | G | H | R | V | F | T | A | Y | fluorescent |
| mKO2 A2G_3_Hc | F | D | L | V | S | H | V | F | C | Y | G | H | R | V | Y | T | A | Y | fluorescent |

**B**

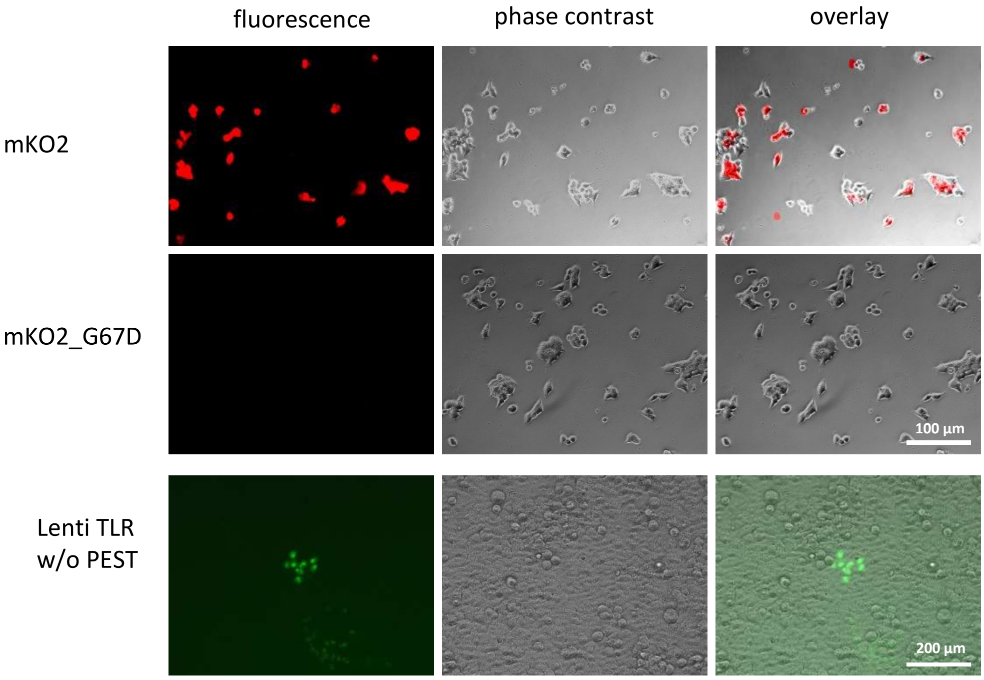


**Supplementary Figure 1: A lentiviral traffic light reporter (TLR) for simultaneous evaluation of gene correction and indels.**
**(A)** Overview on fluorochrome encoding regions of selected known fluorophores. Amino acids aligning with the FCYG fluorochrome of mKO2 are framed in black. In the lower section three cloned and experimentally tested versions of the mKO2 fluorochrome region are depicted. The sequence of mKO2 A2G_Hc_3 served as a basis for the mKO2_mut gene in the TLR vector.
**(B)** Fluorescence microscopic analysis of HEK293-T cells transfected with mKO2 or mKO2_G67D.
**(C)** Fluorescence microscopic analysis of HEK293-T cells transfected with Lenti-TLR construct without PEST signal.

# Supplementary Figure 2

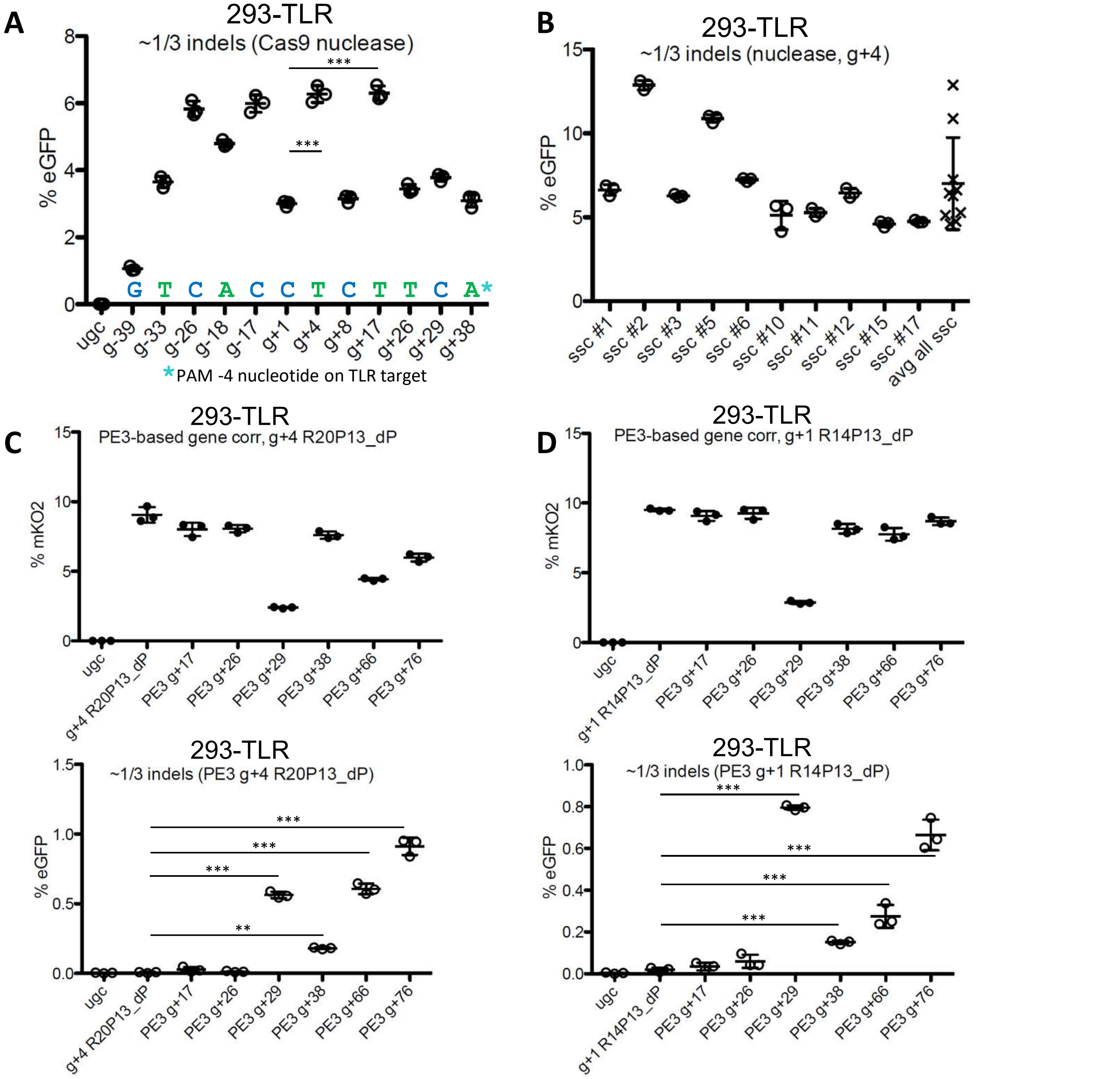


**Supplementary Figure 2: Analysis of a versatile traffic light reporter in HEK-293 cells for simultaneous analysis of gene correction and indel rates.**

**(A)** Analysis of indel formation by different mKO2_mut-targeting sgRNAs in a 293-TLR bulk population. A and T nucleotides at respective PAM -4 positions which can favor +1bp insertions leading to stronger eGFP activation are depicted in green, G and C nucleotides in blue.

**(B)** Analysis of indel formation by Cas9 nuclease with g+4 sgRNA in ten single cell clones (ssc) derived from the 293-TLR bulk population. Open circles depict individual transfections of ssc and tiled crosses on the right hand side mark the averages of single cell clones.

**(C)** Gene correction and indel formation in 293-TLR cells by PE3-based prime editing with g+4 R20P13_dP and different non-edited strand nicking sgRNAs.

**(D)** Gene correction and indel formation in 293-TLR cells by PE3-based prime editing with g+1 R14P13_dP and different non-edited strand nicking sgRNAs.

**(E-I)** see next page

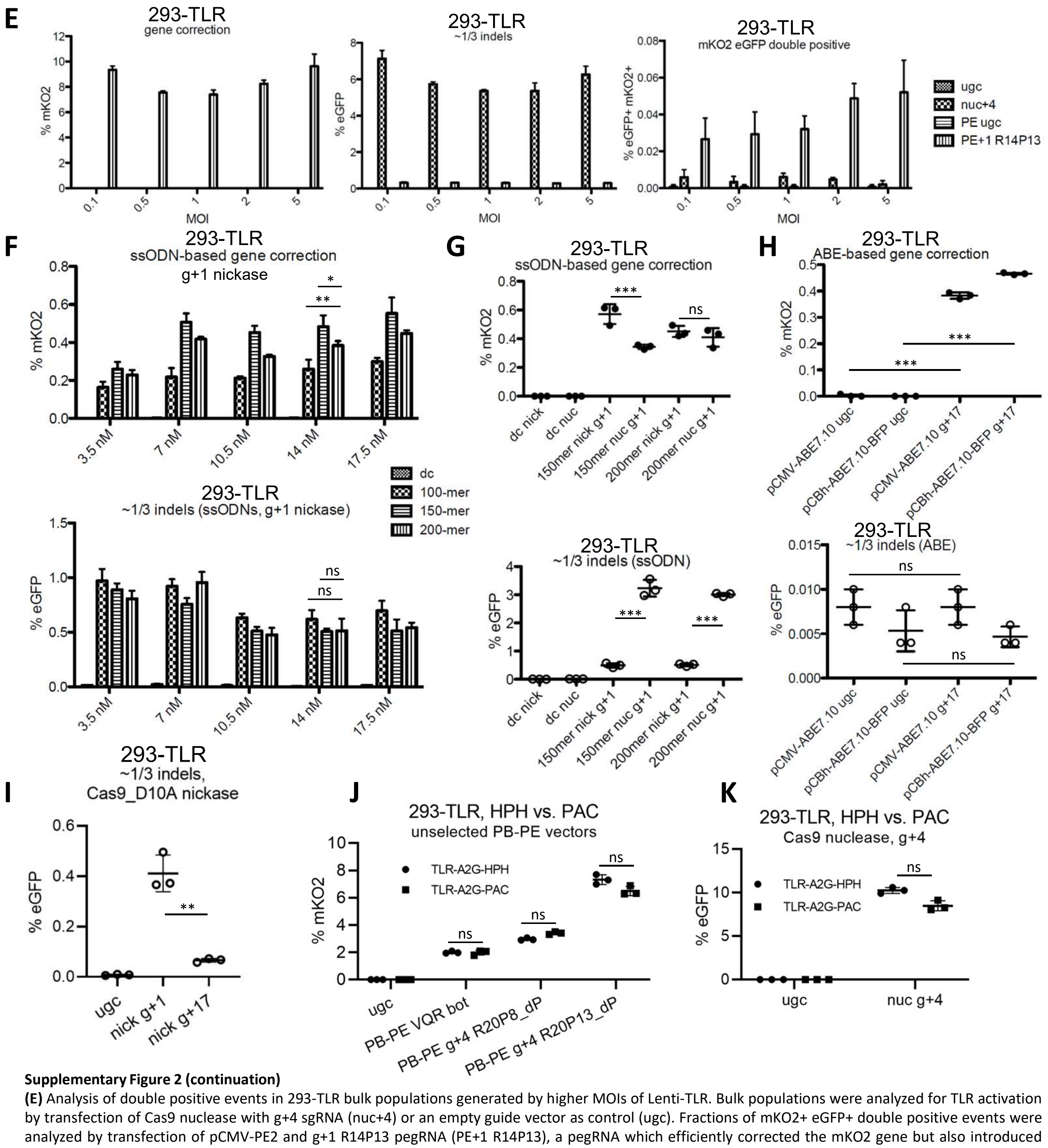


**Supplementary Figure 2 (continuation)**

**(E)** Analysis of double positive events in 293-TLR bulk populations generated by higher MOIs of Lenti-TLR. Bulk populations were analyzed for TLR activation by transfection of Cas9 nuclease with g+4 sgRNA (nuc+4) or an empty guide vector as control (ugc). Fractions of mKO2+ eGFP+ double positive events were analyzed by transfection of pCMV-PE2 and g+1 R14P13 pegRNA (PE+1 R14P13), a pegRNA which efficiently corrected the mKO2 gene but also introduced considerable amounts of indels. Transfection of pCMV-PE2 with an empty pegRNA vector served as unguided control (PE ugc).

**(F)** Analysis of gene correction and indel formation upon transfection of Cas9_D10A nickase and g+1 sgRNA with different concentrations of TLR_A2G+1+17_dP ssODNs of different lengths. Concentrations are given as final in the 350 ul transfection mix applied per well of 12-well. 293-TLR cells transfected with Cas9_D10A, empty guide vector and TLR_A2G+1+17_dP 150-mer served as donor control (dc).

**(G)** FACS analysis of 293-TLR cells transfected with SpCas9 nuclease and SpCas9_D10A nickase and 150-mer or 200-mer gc ssODNs. dc: donor control

**(H)** Analysis of gene correction and indel formation in 293-TLR cells transfected with pCMV-ABE7.10 or newly cloned pCBh-ABE7.10-BFP and g+17 sgRNA. Cells transfected with ABE constructs and empty guide vectors served as unguided controls (ugc).

**(I)** Indel formation by Cas9_D10A nickase with g+1 and g+17 sgRNAs and 14 nM ssODN, ugc: unguided control.

**(J, K)** Comparison of HPH and PAC versions of TLR vectors in HEK-293 cells. MKO2 signal in respective 293-TLR bulk populations was activated by PB-PE vectors (without puromycin selection) **(J)** and eGFP was activated by Cas9 nuclease with g+4 **(K)**.

Data are represented as +/-SD from n=3 and significance was calculated using 1-way ANOVA with Tukey's post-test or 2-way ANOVA with Bonferroni multiple comparisons test for data displayed as groups (****: p≤0.0001; ***: p≤0.001; **: p≤0.01; *: p≤0.05; ns: p>0.05).

# Supplementary Figure 3

```
                                                     GGTGGC        CCATATGTGCCGGATAG           g+1
                                             TGTTACGGCCACCG        GGTATACACGGCC               peg+1 R14P13
GGCGGGCCAATGCCCTTCGCCTTTGACCTGGTGTCCCACGTGTTCTGTTACGACCACCG--------GGTATACACGGCCTATCCGGAGGAGAT mKO2_mut

g+1 nuclease
GGCGGGCCAATGCCCTTCGCCTTTGACCTGGTGTCCCACGTGTTCTGTTACGACCACCGT-------GGTATACACGGCCTATCCGGAGGAGAT ins+1(T)
GGCGGGCCAATGCCCTTCGCCTTTGACCTGGTGTCCCACGTGTTCTGTTACGACCACCG----------------------ATCCGGAGGAGAT del-14
GGCGGGCCAATGCCCTTCGCCTTTGACCTGGTGTCCCACGTGTTCTGTTACGACCACCG----------------------ATCCGGAGGAGAT del-14
GGCGGGCCAATGCCCTTCGCCTTTGACCTGGTGTCCCACGTGTTCTGTTACGACCACCG-------------------------CGGAGGAGAT del-17
GGCGGGCCAATGCCCTTCGCCTTTGACCTGGTGTCCCACGTGTTCTGTTACGACCACCGT-------GGTATACACGGCCTATCCGGAGGAGAT ins+1(T)
GGCGGGCCAATGCCCTTCGCCTTTGACCTGGTGTCCCACGTGTTCTGT----------------------ATACACGGCCTATCCGGAGGAGAT del-14
GGCGGGCCAATGCCCTTCGCCTTTGACCTGGTGTCCCACGTGTTCTGTTACGACCACCGTAACA----------------------GAGGAGAT indel-19+5
GGCGGGCCAATGCCCTTCGCCTTTGACCTGGTGTCCCACGTGTTCTGTTACGACCACCG----------TATACACGGCCTATCCGGAGGAGAT del-2
GGCGGGCCAATGCCCTTCGCCTTTGACCTGGTGTCCCACGTGTTCTGTTACG-------------------------GCCTATCCGGAGGAGAT del-17
GGCGGGCCAATGCCCTTCGCCTTTGACCTGGTGTCCCACGTGTTCTGTT----------------------------------CCGGAGGAGAT del-26

peg+1 R14P13 PE
GGCGGGCCAATGCCCTTCGCCTTTGACCTGGTGTCCCACGTGTTCTGTTACG-------------------------GCCTATCCGGAGGAGAT del-17
GGCGGGCCAATGCCCTTCGCCTTTGACCTGGTGTCCCACGTGTTCTGTTACGACCACCGTA-------------ACGGCCTATCCGGAGGAGAT indel-7+2
GGCGGGCCA-------------------------------------------------------------------------TCCGGAGGAGAT del-65
GGCGGGCCAATGCCCTTCGCCTTTGACCTGGTGTCCCACGTGTT-----ACGGCCACCG--------GGTATACACGGCCTATCCGGAGGAGAT del-5/G67D_corr
GGCGGGCCAATGCCCTTCGCCTTTGACCTGGTGTCCCACGTGTTCTGTTACGGCCACCGGGTC----GGTATACACGGCCTATCCGGAGGAGAT ins+4/G67D_corr
GGCGGGCCAATGCCCTTCGCCTTTGACCTGGTGTCCCACGTGTTCTGTTACGACCACCGTATACACC-GTATACACGGCCTATCCGGAGGAGAT indel-1+8
GGCGGGCCAATGCCCTTCGCCTTTGACCTGGTGTCCCACGTGTTCTGTTACGACCAC----------GGTATACACGGCCTATCCGGAGGAGAT del-2


                                                        GGCCCA        TATGTGCCGGATAGGC         g+4
                                          TTCTGTTACGGCCACCGGGT        ATACACGGCCTAT            peg+4 R20P13
GGCGGGCCAATGCCCTTCGCCTTTGACCTGGTGTCCCACGTGTTCTGTTACGACCACCGGGT--------ATACACGGCCTATCCGGAGGAGAT mKO2_mut

g+4 nuclease
GGCGGGCCAATGCCCTTCGCCTTTGACCTGGTGTCCCACGTGTTCTGTTACGACCACCGGGTA-------ATACACGGCCTATCCGGAGGAGAT ins+1(A)
GGCGGGCCAATGCCCTTCGCCTTTGACCTGGTGTCCCACGTGTTCTGTTACGACCACCGGGTA-------ATACACGGCCTATCCGGAGGAGAT ins+1(A)
GGCGGGCCAATGCCCTTCGCCTTTGACCTGGTGTCCCACGTGTTCTGTTACGACCA-----T--------ATACACGGCCTATCCGGAGGAGAT del-5
GGCGGGCCAATGCCCTTCGCCTTTGACCTGGTGTCCCACGTGTTCTGTTACGACC-------------------------TATCCGGAGGAGAT del-17
GGCGGGCCAATGCCCTTCGCCTTTGACCTGGTGTCCCACGTGTTCTGTTACGACC-------------------------TATCCGGAGGAGAT del-17
GGCGGGCCAATGCCCTTCGCCTTTGACCTGGTGTCCCACGTGTTCTGTTACGACCACC-------------------GCCTATCCGGAGGAGAT del-11
GGCGGGCCAATGCCCTTCGCCTTTGACCTGGTGTCCCACGTGTTCTGTTACGACCACCGGGTA-------ATACACGGCCTATCCGGAGGAGAT ins+1(A)
GGCGGGCCAATGCCCTTCGCCTTTGACCTGGTGTCCCACGTGTTCTGTTACGACCACCGGGTA-------ATACACGGCCTATCCGGAGGAGAT ins+1(A)
GGCGGGCCAATGCCCTTCGCCTTTGACCTGGTGTCCCACGTGTTCTGTTACGACCACCGGGT--------AT-----------CCGGAGGAGAT del-11
GGCGGGCCAATGCCCTTCGCCTTTGACCTGGTGTCCCACGTGTTCTGTTACGACC-------------------------TATCCGGAGGAGAT del-17
GGCGGGCCAATGCCCTTCGCCTTTGACCTGGTGTCCCACGTGTTCTGTTACGACCA-----T--------ATACACGGCCTATCCGGAGGAGAT del-5
GGCGGGCCAATGCCCTTCGCCTTTGACCTGGTGTCCCACGTGTTCTGTTACGACCACCGGGT--------A-----GGCCTATCCGGAGGAGAT del-5
GGCGGGCCAATGCCCTTCGCCTTTGACCTGGTGTCCCACGTGTTCTGTTACGACCACCGGGTA-------ATACACGGCCTATCCGGAGGAGAT ins+1(A)
GGCGGGCCAATGCCCTTCGCCTTTGACCTGGTGTCCCACGTGTTCTGTTACGACCACCGGGTT-------ATACACGGCCTATCCGGAGGAGAT ins+1(T)
GGCGGGCCAATGCCCTTCGCCTTTGACCTGGTGTCCCACGTGTTCTGTTACGACCACCGGGTA-------ATACACGGCCTATCCGGAGGAGAT ins+1(A)


peg+4 R20P13 PE
GGCGGGCCAATGCCCTTCGCCTTTGACCTGGTGTCCCACGTGTTCTGTTACGACCACC----------------ACGGCCTATCCGGAGGAGAT del-8
```

**Supplementary Figure 3: Indel profiles of g+1 and g+4.** Alignments of indels detected after TOPO-TA cloning of mKO2_mut PCR products from transfected and eGFP+ sorted 293-TLR cells. 293-TLR cells were transfected with g+1 or g+4 gRNAs and Cas9 nuclease or with g+1 R14P13 or g+4 R20P13 pegRNAs and pCMV-PE2. Red: insertions and deletions; blue: PAM of respective (pe)gRNA; green: mKO2_mut G67D point mutation [A] or correction thereof [G].

# Supplementary Figure 4

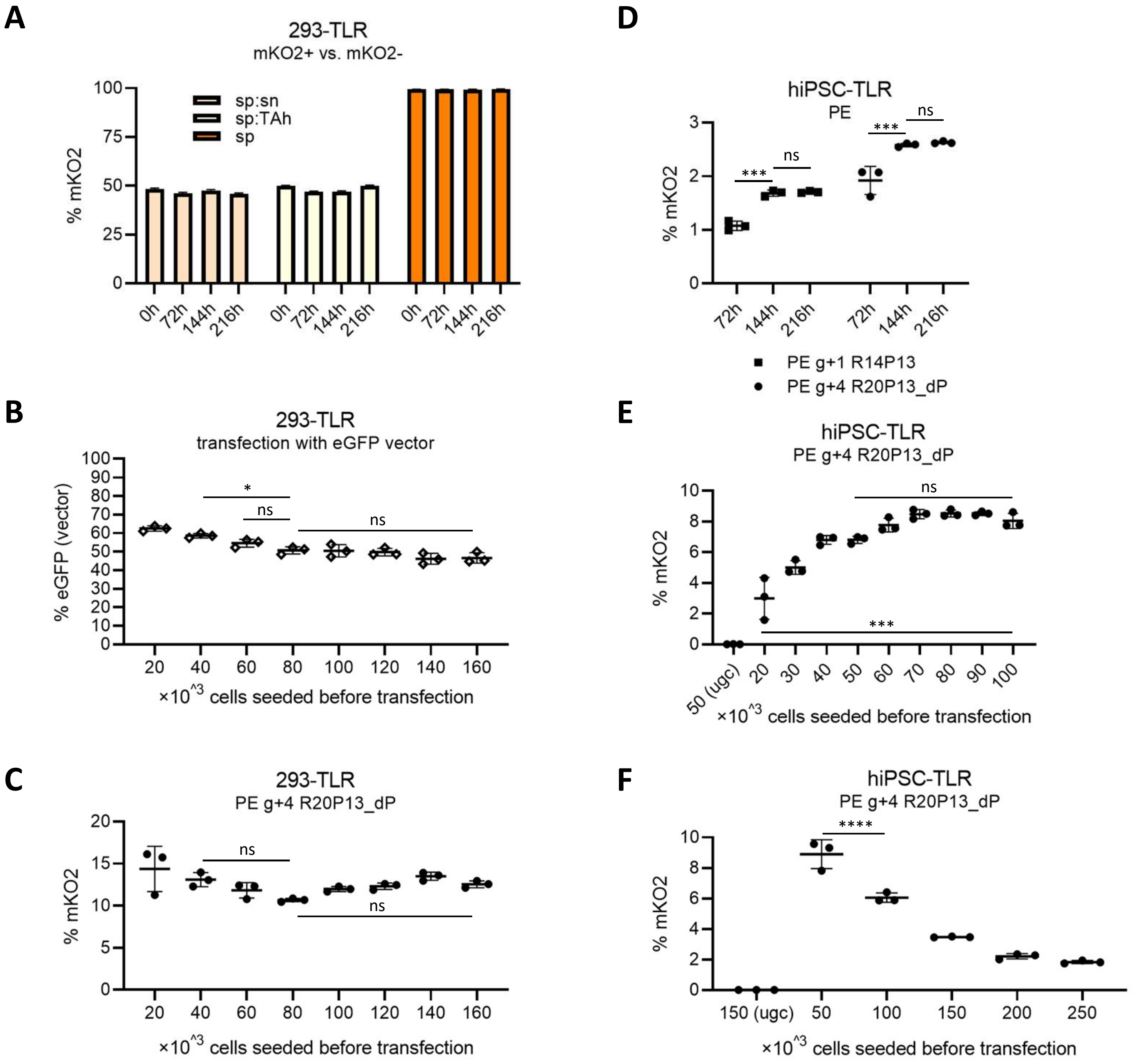


**Supplementary Figure 4: An expressed fluorescent mKO2 transgene does not result in growth disadvantage in 293-TLR. Reporter activity does not decrease over time in hiPSC-TLR but optimal seeding cell numbers are difficult to establish for efficient prime editing.**

**(A)** FACS analysis of 293-TLR cells which were first treated with pCBh-ABE7.10-BFP and g+17 sgRNA and then sorted for the mKO2+ fraction (sp) over a total time of 216h. Those mKO2+ cells were mixed 1:1 either with a sorted mKO2- fraction (sn) or with the original 293-TLR (TAh) and co-cultivated over the same time span.

**(B, C)** Dependency of transfection efficiency (PEI protocol) and prime editing efficiency to number of 293-TLR cells seeded per well of 12-well one day before transfection.

**(D)** FACS analysis for gene corrected mKO2 in hiPSC-TLR cells transfected using Lipofectamine Stem (LFS) reagent with pCMV-PE2 and g+4 R20P13_dP or g+1 R14P13 pegRNAs at three different time points post transfection. Cells were split and cultivated again at each time point of analysis.

**(E, F)** Temporally separated experiments from main Figure 2E, analyzing mKO2 gene correction in hiPSC-TLR cells transfected with pCMV-PE2 and g+4 R20P13_dP pegRNA, with different amounts of cells seeded per well of 12-well one day before transfection.

Data are represented as +/-SD from n=3 and significance was calculated using 1-way ANOVA with Tukey's post-test or 2-way ANOVA with Bonferroni multiple comparisons test for data displayed as groups (****: p≤0.0001; ***: p≤0.001; **: p≤0.01; *: p≤0.05; ns: p>0.05).

# Supplementary Figure 5

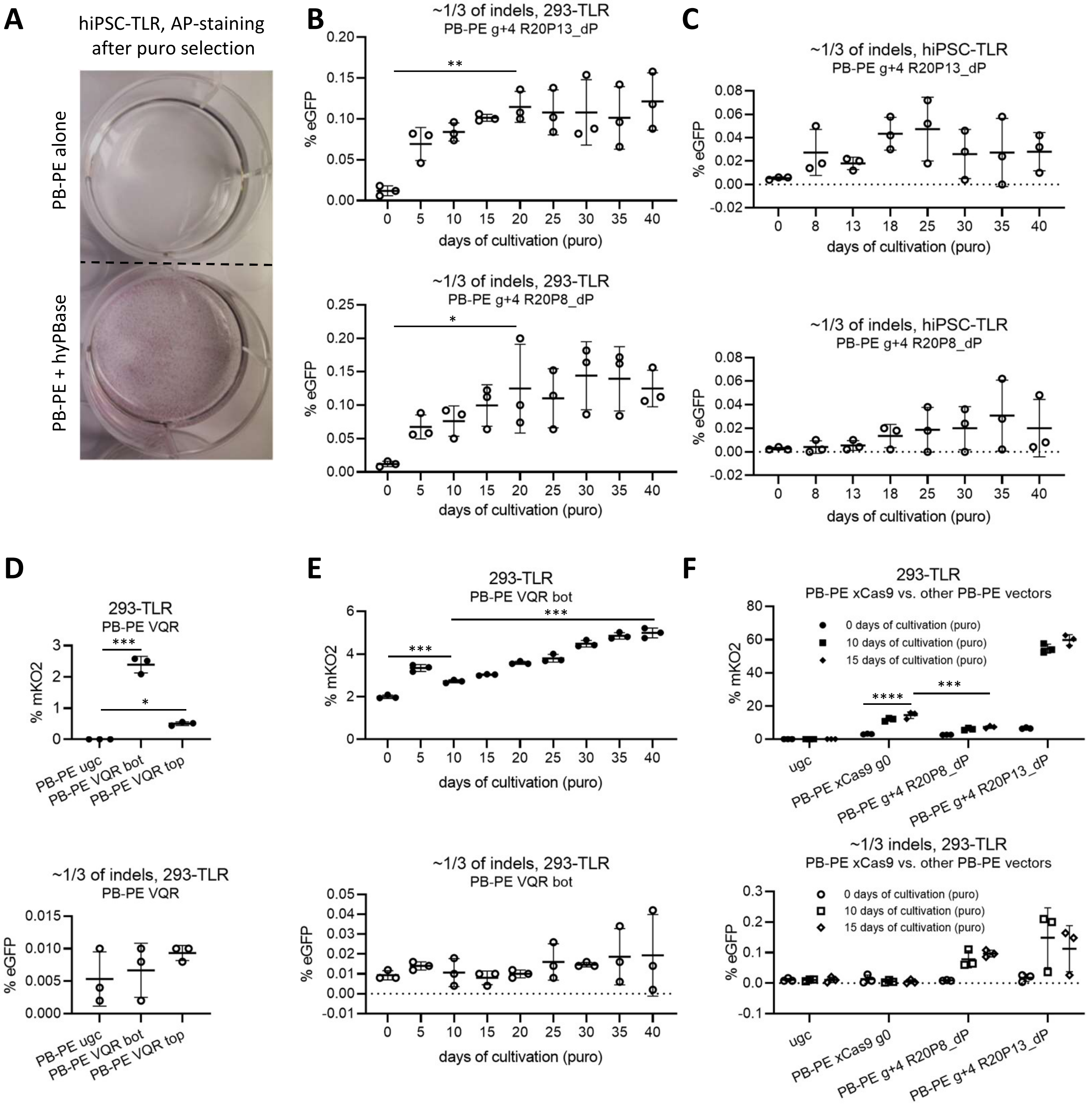


**Supplementary Figure 5: Prime editing using various PB-PE constructs, control experiments and TLR transgene silencing analysis.**

**(A)** Alkaline phosphatase (AP) staining on hiPSC-TLR cells after transfection of PB-PE transposon without or with hyPBase after puromycin selection and expansion.

**(B)** Time-course analysis of indel formation in 293-TLR cells expressing the PB-PE g+4 R20P13_dP (upper panel) or PB-PE g+4 R20P8_dP (lower panel) transposons.

**(C)** Time-course analysis of indel formation in hiPSC-TLR cells expressing the PB-PE g+4 R20P13_dP (upper panel) or PB-PE g+4 R20P8_dP (lower panel) transposons.

**(D)** Gene correction and indel formation in 293-TLR cells by PB-PE VQR bot and PB-PE VQR top, targeting a GGTG and a CGAC motif, respectively, without puromycin selection.

**(E)** Time-course analysis of gene correction and indel formation in 293-TLR cells expressing the PB-PE VQR bot transposon.

**(F)** Time-course analysis of gene correction and indel formation in 293-TLR cells expressing the PB-PE xCas9 g0 transposon vs. other PB-PE transposons.

**(G-I)** see next page

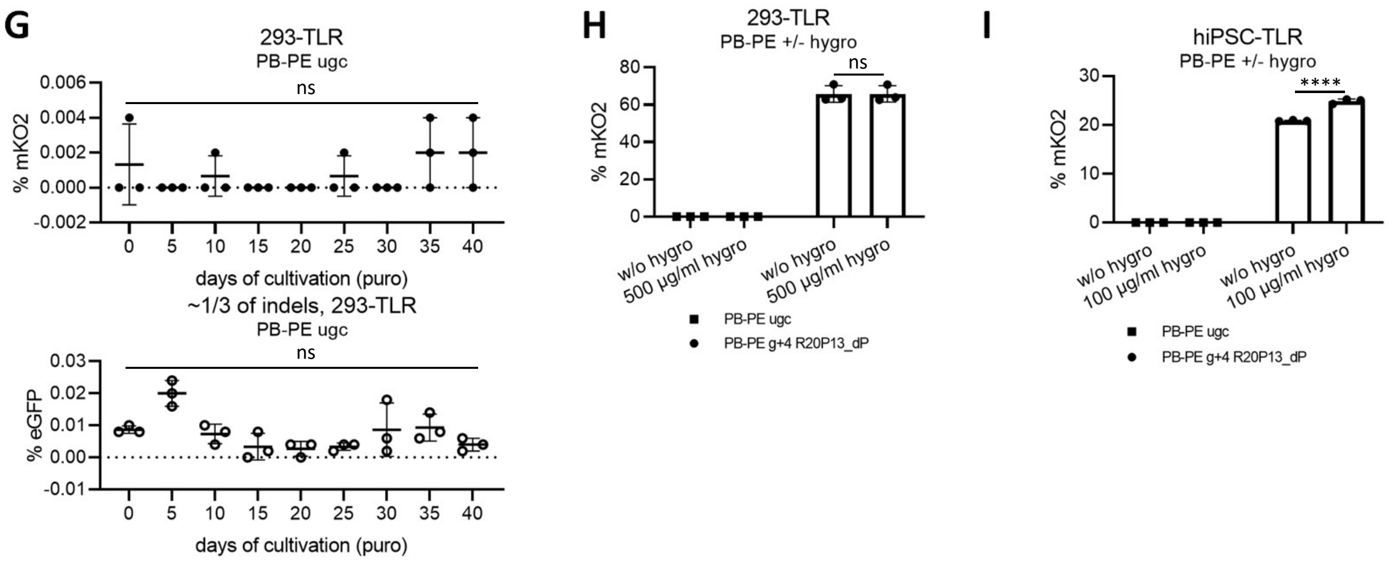


**Supplementary Figure 5 (continuation)**

**(G)** Time-course analysis of gene correction and indel formation in 293-TLR cells expressing the PB-PE transposon without pegRNA guide sequence (ugc, unguided control).

**(H)** Analysis of TLR-silencing in 293-TLR cells during PB-PE gene editing. 293-TLR cells used in PB-PE assays harbor an HPH gene in the TLR cassette, equipping them with resistance to hygromycin B. At day 30 of cultivation, each well of PB-PE transfected cells was split onto two new wells, one containing hygromycin B and one with normal culture medium. Cells were analyzed 5 days later again. Of note, values for category 'w/o hygro' are from the same experiment and correspond to the ones at day 35 of cultivation in main Figure 3E.

**(I)** Analysis of TLR-silencing in hiPSC-TLR cells during PB-PE gene editing. Similar to analysis performed in 293-TLR (Supplementary Figure 5H), cells were split day 8 of cultivation onto two wells (with and without hygromycin B) and analyzed 5 days later again. Values for category 'w/o hygro' are from the same experiment and correspond to ones at day 13 of cultivation in main Figure 3F.

Data are represented as +/-SD from n=3 and significance was calculated using 1-way ANOVA with Tukey's post-test or 2-way ANOVA with Bonferroni multiple comparisons test for data displayed as groups (****: $p \leq 0.0001$; ***: $p \leq 0.001$; **: $p \leq 0.01$; *: $p \leq 0.05$; ns: $p > 0.05$).

# Supplementary Figure 6

A

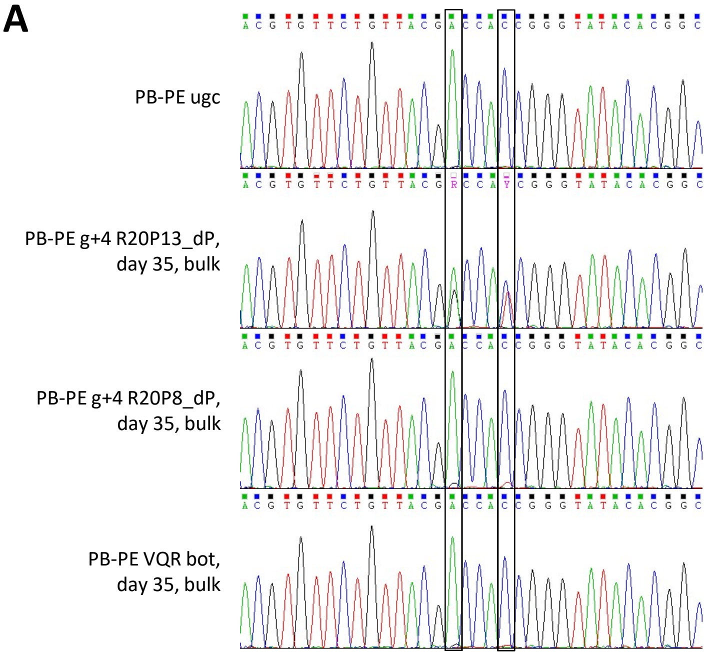


B

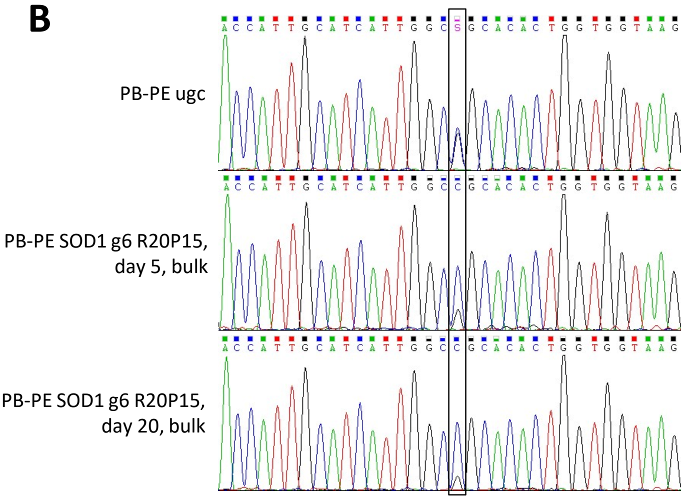


C

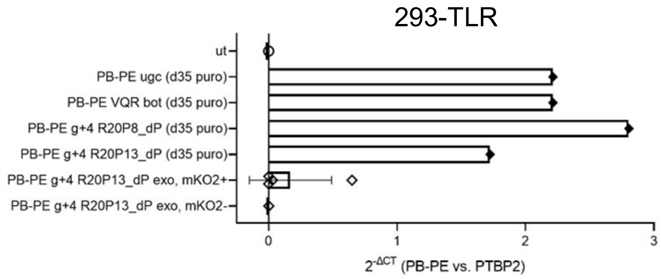


| target | efficiency | slope | intercept |
|---|---|---|---|
| PB-PE | 96.5% | -3.41 | 35.71 |
| PTBP2 | 93.8% | -3.48 | 39.19 |

D

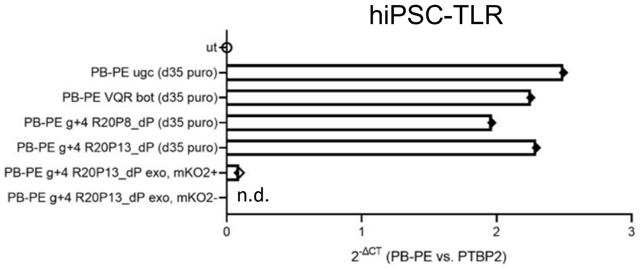


| target | efficiency | slope | intercept |
|---|---|---|---|
| PB-PE | 96.5% | -3.41 | 35.71 |
| PTBP2 | 93.8% | -3.48 | 39.19 |

E

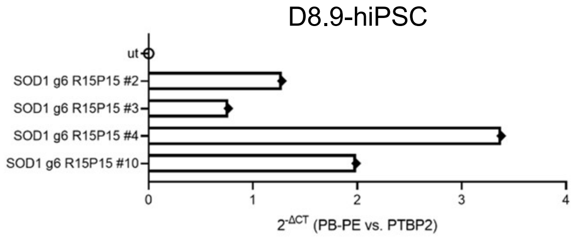


| target | efficiency | slope | intercept |
|---|---|---|---|
| PB-PE | 93.51% | -3.49 | 34.85 |
| PTBP2 | 101% | -3.3 | 36.09 |

**Supplementary Figure 6: Chromatograms of PB-PE gene editing in 293-TLR and D8.9 hiPSC and PB-PE qPCR analysis.**

**(A)** Chromatograms of mKO2 PCR products generated on isolated genomic DNA from PB-PE transfected 293-TLR cells, at day 35 of cultivation. Black framed boxes: locations of G67D point mutation and of g+4 PAM deletion.

**(B)** Chromatograms of SOD1 PCR products generated on isolated genomic DNA from PB-PE SOD1 g6 R20P15 transfected D8.9 hiPSC, at days 5 and 20 of cultivation. PB-PE ugc (unguided control) transfected cells served as controls. Black framed box: location of SOD1_R115G point mutation.

**(C-D)** PB-PE qPCR analysis of transposon containing 293-TLR and hiPSC-TLR bulk populations (day 35 of puromycin selection) as well as excised and FIAU-selected mKO2+ and mKO2- sorted bulk populations originating from PB-PE g+4 R20P13_dP edited population (“exo”). A primer/probe set matching the PTBP2 gene was used as internal reference. qPCR efficiency, slope and intercept was calculated for each reaction set-up and each target using ten-fold serial dilutions of standard plasmids. ut: untreated controls. n.d.: PB-PE signal not detected. Error bar in **(C)** represents ±SD of n = 4 biological replicates. All other values are displayed as mean of n = 3 technical replicates.

**(E)** PB-PE qPCR analysis of gene-corrected and non-excised SOD1 g6 R15P15 clones. Values are displayed as mean of n = 3 technical replicates.

# Supplementary Figure 7

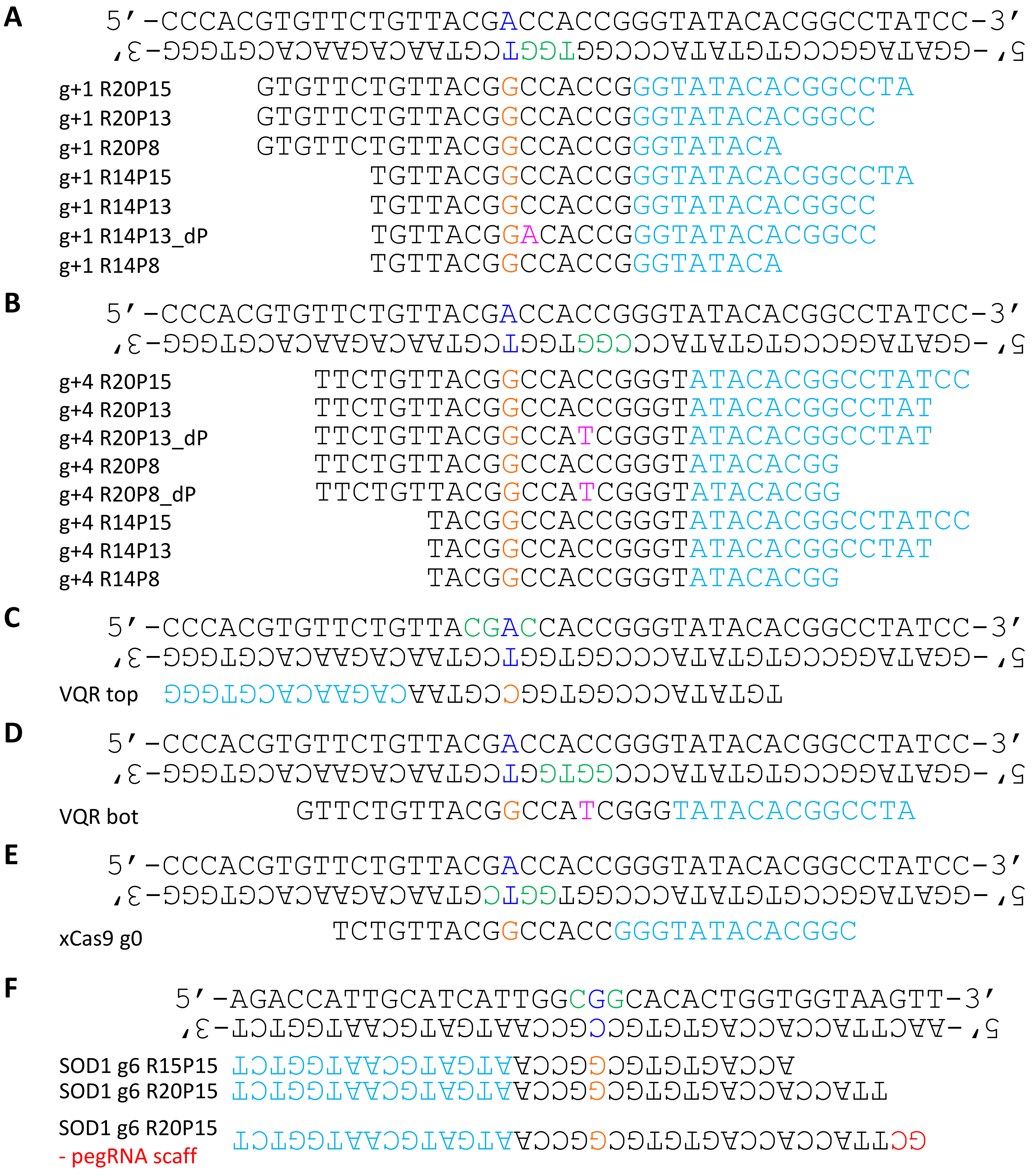


**Supplementary Figure 7: Design and genomic target sites of pegRNAs used in this study.**
**(A, B)** TLR target sequence at the site of the mKO2_G67D point mutation and design of g+1 and g+4 pegRNAs
**(C, D, E)** TLR target sequence at the site of the mKO2_G67D point mutation and design of VQR top and VQR bot and xCas9 g0 pegRNAs
**(F)** SOD1 target sequence at the site of the R115G point mutation and design of SOD1 g6 pegRNAs
Color code: dark blue: to-be edited point mutation; green and in case of PAM-inclusive point mutation dark blue: PAM-site. orange: base for gene correction in pegRNA RTt; pink: PAM deletion in pegRNA RTt in case of PAM exclusive point mutation; light blue: pegRNA PBS; red: part of pegRNA scaffold.
