# Supplementary material for: A selectable all-in-one CRISPR prime editing piggyBac transposon allows for highly efficient gene editing in human cell lines
Source: Sci Rep. 2021 Nov 12;11:22154. doi: 10.1038/s41598-021-01689-2 (PMC8589839; doi:10.1038/s41598-021-01689-2)
Supplement: Supplementary file 2 — Supplementary Information 2. [file 41598_2021_1689_MOESM2_ESM.pdf]

<u>**Supplementary Methods:**</u>

A selectable all-in-one CRISPR prime editing piggyBac transposon allows for highly efficient gene editing in human cell lines

Reto Eggenschwiler[1,2]*, Thomas Gschwendtberger[3,4], Christian Felski[1,2], Christopher Jahn[1,2], Florian Langer[1,2], Jared Sterneckert[5], Andreas Hermann[6,7], Jonathan Lühmann[8], Doris Steinemann[8], Alexandra Haase[2,9,10], Ulrich Martin[2,9,10], Susanne Petri[3,4], Tobias Cantz[1,2,11]*

[1]Research Group Translational Hepatology and Stem Cell Biology, Department of Gastroenterology, Hepatology and Endocrinology, Hannover Medical School, 30625 Hannover, Germany.

[2]REBIRTH-Research Center for Translational Regenerative Medicine, Hannover Medical School, 30625 Hannover, Germany.

[3]Department of Neurology, Hannover Medical School, 30625 Hannover, Germany.

[4]Center for Systems Neuroscience, Hannover Medical School, 30625 Hannover, Germany.

[5]Technische Universität Dresden, Center for Regenerative Therapies Dresden (CRTD), 01307 Dresden, Germany

[6]Translational Neurodegeneration Section „Albrecht-Kossel", Department of Neurology and Center for Transdisciplinary Neurosciences Rostock (CTNR), University Medical Center Rostock, University of Rostock, Rostock, Germany

[7]German Center for Neurodegenerative Diseases (DZNE) Rostock/Greifswald, 18147 Rostock, Germany

[8]Institute of Human Genetics, Hannover Medical School, 30625 Hannover, Germany

[9]Leibniz Research Laboratories for Biotechnology and Artificial Organs (LEBAO), Department of Cardiothoracic, Transplantation and Vascular Surgery, Hannover Medical School, 30625 Hannover, Germany.

[10]Biomedical Research in Endstage and Obstructive Lung Disease (BREATH), German Center for Lung Research (DZL), 30625 Hannover, Germany.

[11]Max Planck Institute for Molecular Biomedicine, Cell and Developmental Biology, 48149 Münster, Germany.

*correspondence: R.E.: eggenschwiler.reto@mh-hannover.de; T.C.: cantz.tobias@mh-hannover.de

Table of contents:

- **Molecular cloning of**
  - TLR constructs **page 2**
  - shRNA and pegRNA expression vectors **page 3**
  - Cas9 and ABE expression vectors **page 3**
  - hyPBase expression vectors **page 3**
  - all-in-one PB-PE vectors **page 4-5**
- **PCRs for molecular cloning page 5**
- **Guidelines for choosing of the (pe)gRNA target sites page 6**
- **Supplementary Table 1: PCR oligos page 7**
- **Supplementary Table 2: direct cloning oligos page 8**
- **Supplementary Table 3: ssODNs for mKO2 gene correction page 9**
- **Supplementary Table 4: qPCR primer and probes page 9**
- **Supplementary Literature page 10**

Molecular cloning

<u>TLR constructs</u>: A 675 bp amplicon containing the mKO2 sequence was generated using 'mKO2-hCdt1(30/120) / pCSII-EF' (FUCCI-Red[1]) as template with *mKO2 AgeI for* and *mKO2 BsrGI-T2A rev* primers and inserted into 'Lenti-CG2AP'[2] digested with AgeI and BsrGI, thereby replacing eGFP with mKO2. Sanger sequencing of a picked bacterial clone revealed an mKO2_G67D point mutation and this plasmid was denoted 'Lenti-CKO2AP_G67D'. A 540 bp PCR amplicon was then generated using Lenti-CKO2AP_G67D as template with *mKO2 MluI G67 corr* and *mKO2 BsrGI-T2A rev* primers and inserted into Lenti-CKO2AP_G67D digested with MluI and BsrGI, thereby replacing mKO2_G67D with mKO2, resulting in 'Lenti-CKO2AP'. Three 169 bp sequences harboring different mKO2 mutations were ordered as gene synthesis (Eurofins Genomics, Ebersberg, Germany), denoted as 'pEX-A128 TLR A2G_Hc_gs', 'pEX-A128 TLR A2G_2_Hc_gs' and 'pEX-A128 TLR A2G_3_Hc_gs', and cloned into Lenti-CKO2AP digested with MluI and BstBI, resulting in 'Lenti-CKO2AP_A2G_Hc', 'Lenti-CKO2AP_A2G_2_Hc' and 'Lenti-CKO2AP_A2G_3_Hc'. A 533 bp gene synthesis of an mKO2_G67D gene with removed frame-shifted STOP codons was ordered, denoted as 'pEX-A2-mKO2_G67D del STOP' and ligated into Lenti-CKO2AP_G67D digested with MluI and BsrGI, thereby replacing mKO2_G67D with mKO2_G67D del STOP, resulting in 'Lenti-G67D -STOP'. An 829 bp PCR amplicon was generated using Lenti-CGIP[3] as template with *SexAI-P2AG+2 for* and *eGFP NsiI rev* primers and inserted into Lenti-G67D –STOP, thereby adding a +2bp-frameshifted eGFP after the PAC gene, resulting in 'Lenti-TLR'. An 829 bp PCR amplicon was then generated using Lenti-TLR as template with *del GFP_alt_ATG for* and *eGFP NsiI rev* primers and inserted into Lenti-G67D –STOP, thereby adding a +2bp-frameshifted eGFP without START codon after the PAC gene, resulting in 'Lenti-TLR-GFP_del_ATG'. A 404 bp PCR amplicon was generated using Lenti-TLR as template with *mKO MluI for* and *mKO das 2 rev* primers (PCR A) and a 611 bp PCR was generated using Lenti-TLR as template with *mKO das 1 for* and *puroR BstEII rev* primers (PCR B). PCR B served then as template, whereas PCR A was employed as mega-primer for generation of a 748 bp amplicon with *puroR BstEII rev*, resulting in PCR C. PCR B was digested using Van91I and resulting 155 bp fragment served as mega-primer for amplification of a 292 bp fragment using Lenti-TLR as template and *mKO MluI for* as primer, resulting in PCR D. PCR C served then as template, whereas PCR D was employed as mega-primer for generation of a 748 bp amplicon with *puroR BstEII rev* primer, which was then TOPO-ligated into pCR2.1-TOPO, resulting in 'TOPO mKO2_dATG'. TOPO mKO2_dATG was digested using MluI-HF and BstEII and inserted into 'Lenti-TLR-GFP_del_ATG' digested with the same enzymes, thereby removing +2bp-frameshifted START codons in mKO2_G67D del STOP, resulting in 'Lenti-TLR_dATG'. A 570 bp PCR amplicon was generated using Lenti-TLR_dATG as template with *puro BstEII for* and *PEST puro rev* primers (PCR E) and an 831 bp PCR was generated using Lenti-TLR_dATG as template with *PEST T2A eGFP for* and *eGFP NsiI rev* primers (PCR F). PCR E served then as template, whereas PCR F was employed as mega-primer for generation of a 1377 bp amplicon with *puro BstEII for*, which was then digested using BstEII and NsiI and ligated into Lenti-TLR_dATG digested with the same enzymes, thereby introducing a +2bp-frameshifted PEST sequence in between PAC and +2bp-frameshifted eGFP, resulting in 'Lenti-TLR-PEST'. A 169 bp sequence harboring an mKO_A2G sequence was ordered as gene synthesis, denoted as 'pEX-A128-TLR_A2G_3_gs', and cloned into Lenti-TLR-PEST digested with MluI and BstBI, thereby changing mKO2_G67D del STOP to mKO2_mut resulting in 'Lenti-TAp' (lab internal reference: Lenti-TLR_A2G). A 176 bp PCR amplicon was generated using Lenti-TAp as template with *HiFi PEST for* and *HiFi T2A_dB rev* primers (PCR G) and a 788 bp PCR was generated using Lenti-TAp as template with *HiFi T2A_dB for* and *HiFi eGFP rev* primers (PCR H). PCRs G and H were then used together with Bsu36I and NsiI-HF digested Lenti-TAp for NEB HiFi ligation (New England BioLabs #E2621L), thereby removing a BmgBI site in the T2A site between PEST and eGFP, resulting in 'Lenti-TLR-A2G_dB'. A 1112 bp gene synthesis of the HPH gene was ordered with synonymous codons replacing +2bp-framshifted START and STOP signals in the HPH sequence, denoted as 'pEX-A258-hygR_daS_gs'. A 1112 bp PCR amplicon was then generated using pEX-A258-hygR_daS_gs as template with *HiFi T2A for* and *HiFi PEST-hygR rev* primers and inserted into Lenti TLR

A2G_dB digested with BmgBI and Bsu36I by NEB HiFi ligation, thereby replacing PAC with HPH, resulting in 'Lenti-TAh' (lab internal reference: Lenti-TAh_daS).

sgRNA and pegRNA expression vectors: A 178 bp sequence harboring part of the U6 promoter and an sgRNA scaffold interrupted by two BbsI-sites was ordered as gene synthesis, denoted as 'pEX-A2-pU6-BbsI-scaffold', and cloned into 'pEX-A2 TTR gRNA 2-24'[3] digested with NdeI and XbaI, thereby replacing TTR gRNA 2-24 sequence with two BbsI sites, resulting in 'pU6-BbsI'. This plasmid served as parental construct for all sgRNA vectors used in this publication and descendants were generated by digestion using BbsI-HF enzyme and subsequent ligation of annealed (+) and (-) oligos harboring CRISPR protospacer sequences (see Supplementary Table 2). A 188 bp sequence harboring part of the U6 promoter and pegRNA scaffold interrupted by two BbsI-sites and followed by two BsaI-sites was ordered as gene synthesis, denoted as 'pEX-A128-pegRNA(BB)scaff_gs', and cloned into pU6-BbsI digested with NdeI and XbaI, thereby replacing the sgRNA scaffold with the pegRNA scaffold, resulting in 'pU6-pegRNA(BB)'. This plasmid served as parental construct for all pegRNA expression vectors used in this publication and descendants were generated by first inserting annealed oligos harboring protospacer sequences into BbsI digested pU6-pegRNA(BB) and subsequent digestion of resultant vectors with BsaI-HFv2 for insertion of annealed oligos harboring RTt-PBS sequences (see Supplementary Table 2).

Cas9 and ABE expression vectors: A 174 bp PCR amplicon was generated using 'pU6-(BbsI)_CBh-Cas9-T2A-BFP'[4] as template with *CBh AgeI for* and *mamD10A mut rev* primers (PCR I) and a 245 bp PCR was generated using pU6-(BbsI)_CBh-Cas9-T2A-BFP as template with *mamD10A mut for* and *mamCas9 BglII rev_1* primers (PCR J). PCRs I and J served then as template for generation of a 390 bp amplicon with *CBh AgeI for* and *mamCas9 BglII rev_1*, which was then TOPO-ligated into pCR2.1-TOPO, resulting in 'TOPO-mamCas9_D10A_mut'. TOPO-mamCas9_D10A_mut was digested using AgeI and BglII and a 379 bp fragment harboring the Cas9_D10A mutation was ligated into pU6-(BbsI)_CBh-Cas9-T2A-BFP digested with the same enzymes, thereby converting Cas9 to Cas9_D10A, resulting in 'Cas9_D10A-BFP'. A 695 bp PCR amplicon was generated using 'pCMV-ABE7.10'[5] as template with *TadA_cc NotI for* and *TadA 7.10 XhoI rev* primers and inserted into pCMV-ABE7.10 digested with NotI and XhoI, thereby introducing silent codon changes into the beginning of the TadA gene sequence, resulting in 'pCMV-ABE7.10_cc'. A 1242 bp PCR amplicon was then generated using pCMV-ABE7.10_cc as template with *TadA AgeI for_3* and *D10A fus rev* primers and TOPO-ligated into pCR2.1-TOPO, resulting in 'TOPO TadA PCR1'. Furthermore, a 256 bp PCR amplicon was generated using Cas9_D10A-BFP as template with *D10A fus for* and *mamCas9 BglII rev_2* primers and TOPO-ligated into pCR2.1-TOPO, resulting in 'TOPO TadA PCR2'. A 1331 bp PCR amplicon was then generated using TOPO TadA PCR1 as template with *T7e* and *D10A fus rev* primers (PCR K) and a 346 bp PCR amplicon was generated using TOPO TadA PCR2 as template with *D10A fus for* and *M13 rev* primers (PCR L). PCRs K and L were then used together with ApaI and HindIII-HF digested TOPO TadA PCR1 for NEB HiFi ligation, resulting in 'TOPO TadA-D10A fus'. TOPO TadA-D10A fus was digested using AgeI-HF and BglII and a 1444 bp fragment was ligated into Cas9_D10A-BFP, thereby adding a TadA-TadA*(7.10) CDS in front of Cas9_D10A, resulting in 'pCBh-ABE7.10-BFP' (lab internal reference: TadA-D10A-BFP).

hyPBase expression vectors: Lenti-CKO2AP was digested using EcoRI-HF and NdeI and a 1470 bp fragment encompassing the chicken-β-actin promoter rabbit globin intron sequence was cloned into 'pCMV-hyPBase'[6] digested with the same enzymes, thereby replacing the CMV promoter with a CMV i/e enhancer – chicken β-actin – rabbit globin intron (CAG) promoter[7], resulting in 'pCAG-hyPBase'. A 739 bp sequence of the hyPBase gene harboring R372A, K375A and D450N mutations[8] was ordered as gene synthesis, denoted as 'pEX-A128-hyPBase_exo gs', and cloned into pCMV-hyPBase digested with KasI and BsrGI-HF, thereby introducing above mutations into the hyPBase gene, resulting in 'pCMV-hyPBase_exo'. Lenti CKO2AP was digested using EcoRI-HF and NdeI and a 1470 bp fragment encompassing the chicken-β-actin promoter rabbit globin intron sequence was cloned into pCMV-hyPBase_exo, thereby replacing the CMV promoter with a CAG promoter, resulting in 'pCAG-

hyPBase_exo'. The sequence of the 739 bp gene synthesis of the hyPBase gene harboring R372A, K375A and D450N mutations is given below, where codons of mutated amino acids are highlighted in red and sites for restriction enzymes which are compatible for direct subcloning into pCMV-hyPBase are written in blue.

GGCGCCCACCTGACCATCGACGAGCAGCTGCTGGGCTTCAGGGGCAGGTGCCCCTTCAGGGTCTATATCCCCAACAAGCCCAG
CAAGTACGGCATCAAGATCCTGATGATGTGCGACAGCGGCACCAAGTACATGATCAACGGCATGCCCTACCTGGGCAGGGGC
ACCCAGACCAACGGCGTGCCCCTGGGCGAGTACTACGTGAAGGAGCTGTCCAAGCCCGTCCACGGCAGCTGCAGAAACATCA
CCTGCGACAACTGGTTCACCAGCATCCCCCTGGCCAAGAACCTGCTGCAGGAGCCCTACAAGCTGACCATCGTGGGCACCGTG
GCCAGCAACGCCAGAGAGATCCCCGAGGTCCTGAAGAACAGCAGGTCCAGGCCCGTGGGCACCAGCATGTTCTGCTTCGACG
GCCCCCTGACCCTGGTGTCCTACAAGCCCAAGCCCGCCAAGATGGTGTACCTGCTGTCCAGCTGCGACGAGGACGCCAGCATC
AACGAGAGCACCGGCAAGCCCCAGATGGTGATGTACTACAACCAGACCAAGGGCGGCGTGGACACCCTGAACCAGATGTGCA
GCGTGATGACCTGCAGCAGAAAGACCAACAGGTGGCCCATGGCCCTGCTGTACGGCATGATCAACATCGCCTGCATCAACAG
CTTCATCATCTACAGCCACAACGTGAGCAGCAAGGGCGAGAAGGTGCAGAGCCGGAAAAAGTTCATGCGGAACCTGTACA

In case of problems of cloning pCAG-hyPBase_exo, using SPB-002 PBx (encoding 'excision-only' piggyBac transposase) from HeraBiolabs (Lexington KY, US) is recommended. However, SPB-002 PBx is driven by CMV promoter instead of CAG. The CAG promoter has been used shown to be highly efficient and more resistant to transgene silencing for applications in human and mouse pluripotent cell lines[3].

__All-in-one PB-PE vectors__: pU6-pegRNA(BB) was digested using BbsI-HF and annealed *BbsI-SacI-BbsI (+)* and *BbsI-SacI-BbsI (-)* oligos were ligated into the vector, thereby adding a SacI restriction enzyme site in between the two BbsI sites, resulting in 'pU6-pegRNA BbsI 12sp'. pU6-pegRNA BbsI 12sp was then digested using BsaI-HFv2 and annealed *BsaI-AhdI-BsaI (+)* and *BsaI-AhdI-BsaI (-)* oligos were ligated into the vector, thereby adding an AhdI restriction enzyme site in between the two BsaI sites, resulting in 'pU6-pegRNA (B12B)'. A 385 bp PCR amplicon was generated using 'AAT-PB-CG2APtk'[3] as template with *5'PB ApoI for* and *5'PB-U6 StyI rev* primers, digested with StyI-HF and ApoI-HF and inserted into pU6-pegRNA (B12B) digested with StyI-HF + MfeI-HF, thereby adding the 5' piggyBac recognition motif (5'PB) before the U6 promoter, resulting in '5'PB-pU6(BB)'. A 316 bp PCR amplicon was then generated using AAT-PB-CG2APtk as template with *MCS-3'PB XbaI for* and *3'PB PciI rev* as primers and TOPO-ligated into pCR2.1-TOPO, resulting in 'TOPO 3'PB-MCS'. TOPO 3'PB-MCS was then digested using PciI and XbaI and a 304 bp fragment was introduced into 5'PB-pU6(BB) digested with the same enzymes, thereby adding a multiple cloning site (MCS) and a 3' piggyBac recognition motif (3'PB) downstream of the pegRNA cloning site, resulting in 'PB-pU6(BB)-MCS'. AAT-PB-CG2APtk was digested with EcoRI-HF and NheI-HF and a 1725 bp fragment encompassing a CAG promoter sequence was introduced into PB-pU6(BB)-MCS digested with MfeI-HF and NheI-HF, thereby adding a CAG promoter in between the pegRNA cloning site and the MCS, resulting in 'PB-pU6(BB)-CAG'. A 1372 bp PCR amplicon was generated using AAT-PB-CG2APtk as template with *pTK_dBamHI for* and *bGH_dBbsI AscI rev* primers and TOPO-ligated into pCR2.1-TOPO, resulting in 'TOPO P2A-pTK PCR1'. A 608 bp PCR amplicon was generated using AAT-PB-CG2APtk as template with *P2A pTK EcoRI for* and *pTK_dBsaI rev* primers and TOPO-ligated into pCR2.1-TOPO, resulting in 'TOPO P2A-pTK PCR2'. A 1461 bp PCR amplicon was then generated using TOPO P2A-pTK PCR1 as template with *T7e* and *pTK_dBamHI for* primers and a 699 bp PCR amplicon was generated using TOPO P2A-pTK PCR2 as template with *pTK_dBsaI rev* and *M13 rev* primers and NEB HiFi-ligated into TOPO P2A-pTK PCR2 digested with ApaI and HindIII-HF, resulting in 'TOPO P2A-pTK-pA_dBBB'. TOPO P2A-pTK-pA_dBBB was then digested with AscI and EcoRI-HF and a 1948 bp fragment encompassing a P2A site, a PAC-thymidine kinase fusion gene and a bGH poly-A site without BsaI, BamHI or BbsI restriction sites (P2A-pTK-pA_dBBB), was inserted into PB-pU6(BB)-CAG digested with the same enzymes, thereby adding P2A-pTK-pA_dBBB in between the CAG promoter and the 3'PB site, resulting in 'PB-pU6(BB)-CpTK'.

Important: from this point on, all further cloning steps were performed using NEB Stable competent E.coli (New England Biolabs #C3040H) at 30°C incubation temperature and following manufacturer's instructions. In our experience, other competent cells were mostly unable to handle the large and complex plasmid constructs. We strongly recommend following this protocol for efficient sub-cloning of all PB-PE constructs!

pCMV-PE2[9] was digested using BamI-HF, NotI-HF and EcoRI-HF and a 4215 bp fragment encompassing Cas9_H840A was ligated into PB-pU6(BB)-CpTK digested with BamHI-HF and NotI-HF, thereby adding a Cas9_H840A CDS in between the CAG promoter and the P2A site, resulting in 'PB-pU6(BB)-CH840ApTK'. A 2151 bp sequence harboring a 33 amino acid linker and an M-MLV reverse transcriptase sequence without BsaI, BamHI or BbsI restriction sites (MMLV-RT_dBBB) was ordered as gene synthesis, denoted as 'pEX-A258 MMLV-RT_dBBB_gs', and cloned into PB-pU6(BB)-CH840ApTK digested with BamHI-HF and EcoRI-HF, thereby adding MMLV-RT_dBBB in between Cas9_H840A and P2A, resulting in 'PB-PE'. A 792 bp PCR amplicon was generated using lenti-Cas9-VQR-Blast[10] as template with *VQR SanDI for* and *VQR BamHI rev* primers and NEB HiFi-ligated into PB-PE digested with FD-KflI (Thermo Fisher Scientific #FD2164) and BamHI-HF, thereby replacing CDS of key amino acids in Cas9_H840A to generate Cas9_H840A_VQR, resulting in 'PB-PE VQR'. For generation of PB-PE xCas9, a 2240 bp gene synthesis containing a fragment of SpCas9 with A262T, R324L, S409I, E480K, E543D and M694I mutations was ordered (denoted as pEX-A258-TLIKDI_gs). The gene synthesis spanned a bit more than 20 bp over the two existing PflMI sites in PB-PE and was flanked by additional BbsI sites which would then cut inside those 20 bp. pEX-A258-TLIKDI_gs was digested with BbsI-HF yielding a 2221 bp fragment, which was then NEB HiFi ligated into PB-PE digested with PflMI, resulting in 'PB-PE_TLIKDI'. A 311 bp amplicon was generated using PB-PE as template with *xCas9 HiFi for* and *E1219V rev* primers and a 504 bp amplicon was generated with the same template and *E1219V for* and *xCas9 HiFi rev* primers. Those two PCR products were then NEB HiFi ligated into PB-PE_TLIKDI digested with FD-KflI (Thermo Fisher Scientific #FD2164), resulting in 'PB-PE xCas9'.

PB-PE, PB-PE VQR or PB-PE xCas9 vectors were digested using BbsI-HF and BsaI-HFv2 and annealed oligos containing the respective protospacers, pegRNA scaffold and RTt-PBS sequences (see Supplementary Table 2) were ligated into the corresponding vectors to generate 'PB-PE g+4 R20P8_dP', 'PB-PE g+4 R20P13_dP', 'PB-PE VQR top', 'PB-PE VQR bot', 'PB-PE xCas9 g0', 'PB-PE SOD1 g6 R15P15' and 'PB-PE SOD1 g6 R20P15'.

Maxi preps of all PB-PE vectors were Sanger sequenced using primers spanning the entire expression cassettes in order to ensure fidelity of plasmid amplification in NEB Stable competent E.coli.

All restriction enzymes were purchased from New England BioLabs and used according to manufacturer's instructions, unless indicated otherwise.

PCRs for molecular cloning

All PCRs were performed using Phusion™ Hot Start II High-Fidelity DNA-Polymerase (Thermo Fisher Scientific #F549L) with 0.25 ng/µl of template DNA, 0.2 µM of each primer and 0.2 mM of each, dATP, dGTP, dCTP and dTTP. PCRs on amplicons with high GC-content (>60%) were performed in GC-buffer in cases where amplification was weak or unsuccessful in HF-buffer. For PCRs involving megaprimers or primers with strong secondary structure / dimer formation potential, 5% DMSO was added. All PCRs were established by an annealing temperature gradient ranging from -3°C of the primer pair's lowest until +5°C of the primer pair's highest annealing temperature, but maximally 70°C. Annealing temperatures were calculated using Thermo Fisher Tm calculator for Phusion™ polymerases (https://www.thermofisher.com/de/de/home/brands/thermo-scientific/molecular-biology/molecular-biology-learning-center/molecular-biology-resource-library/thermo-scientific-web-tools/tm-calculator.html). Sequences of primer oligos used for PCRs can be found in Supplementary Table 1.

Guidelines for choosing of the (pe)gRNA target sites

TLR vector target sites: As described in Results part and shown in Figure 1B, we had purposely introduced additional NGG PAMs into the TLR vector sequence which we then later on used as gRNA and pegRNA targeting locations. While the original mKO2 sequence already provided a natural targeting location for g+1 (pe)gRNAs, we intentionally introduced another one just 3 bases downstream along the mKO2 sequence, thereby providing a targeting location for g+4 (pe)gRNAs. Generally, the pCMV-PE2 vector originally published in 2019 by A. Anzalone and colleagues[9] can only modify genomic sequences starting from position -3 upstream of the targeted PAM sequence, i.e. 5'-N_{-3},N_{-2},N_{-1},N_{PAM},G_{PAM},G_{PAM},N_{+1},N_{+2},N_{+3},N_{+4}…N_{+n}-3'. The reason behind this is that the SpCas9_H840A nickase nicks the DNA in between position -4 and position -3 upstream of the PAM. This defines the range which can be edited using a prime editor with a pegRNA which binds at a certain targeting location. In the context of the TLR this means that the locations at g+1 and g+4 were the only ones in close proximity of the G67D point mutation and within the editable range of PE vectors. For example, a pegRNA binding at the g+8 position of the TLR would not be amenable for prime editing of G67D, because the DNA would be nicked 3 bases downstream from the G67D and the reverse transcriptase would extend the sequence from thereon further downstream. In this case an edit encoded by the pegRNA would not be installed.

Additional NGG PAMs for potential prime editing in the TLR are rather distant (nick positions at -27 or +23 bp) from the G67D point mutation and we did not analyze if they could be employed for efficient editing. However, the work from Anzalone and culleagues showed that the editing efficiency decreased with higher distances in between the pegRNA binding site to the point mutation and we would expect similar findings in our context[9]. Nevertheless, we chose additional targeting locations for the PB-PE VQR and PB-PE xCas9 vectors in close proximity to G67D. This was possible because those vectors can target other PAMs than NGG.

Regarding the pegRNA primer binding site (PBS) and reverse transcriptase template (RTt), we geared to the designs which were successfully used earlier[9]. As outlined in Results and shown in Supplementary Figure 7, we chose RTt lengths of 14 and 20 nucleotides, respectively and PBS lengths of 8, 13 and 15 nucleotides and we tested all six combinations of them for both, g+1 and g+4 targeting pegRNAs.

SOD1 target site: For editing of the SOD1_R115G point mutation, we had two choices of targeting locations nearby. We used the 'SOD1 g6' location, because doing so allowed us to simultaneously edit the R115G mutation and disrupt the PAM sequence. This is due to the nature of the C>G SNP resulting in R115G also resulting in introduction of an additional PAM into the affected allele which was not present in the wild type allele (Supplementary Figure 7). We call this type of point mutation 'PAM inclusive'. The design of a pegRNA specifically targeting the affected allele worked so well in our hands (Figure 5A) that we did not evaluate other targeting locations. For more mechanistic studies, evaluation of the second PAM three nucleotides upstream of SOD1 g6 could be interesting in the future.

Supplementary Table 1: PCR oligos

oligo name	oligo sequence
3'PB PciI rev	TAAACATGTTTAACCCTAGAAAGATAATCATATTGTGAC
5'PB ApoI for	ATAAAATTTGTTAACCCTAGAAAGATAGTCTGCG
5'PB-U6 StyI rev	AAACCTTGGTACCGGGTCCAGTCGACTGAATTGGTTCCTTTAAAGCCTGCTTTTTTGTACATGATATCTATAACAAGAAAATATATATATAATAAGTTATCACG
bGH_dBbsI AscI rev	TATGGCGCGCCCCATAGAGCCCACCGCATCCCCAGCATGCCTGCTATTGTCCTCCCAATCCTC
CBh AgeI for	TGGACCGGTGCCACCATGGAC
D10A fus for	GATAAAAAGTATTCTATTGGTTTAGCCATCGGCACCAACTCTGTG
D10A fus rev	CACAGAGTTGGTGCCGATGGCTAAACCAATAGAATACTTTTTATC
del GFP_alt_ATG for	AAAACCTGGTGCATGACCCGCAAGCCCGGTGCCGGCTCCGGAGAGGGCCGGGGCTCTCTGCTGACCTGTGGCGACGTGGAGGAGAACCCCGGCCCTCCAAgTGGTGAGCAAGGGCGAGG
E1219 for	CCTCTGCCGGCGTGCTGCAGAAGGGA
E1219 rev	TCCCTTCTGCAGCACGCCGGCAGAGG
eGFP NsiI rev	TTTATGCATTTACTTGTACAGCTCGTCCATGCC
HiFi eGFP rev	CCCTCGAGGTCGACGGTATC
HiFi PEST for	AAAGCCACGGCTTCCCC
HiFi PEST-hygR rev	CATCGTCCTGAGCGGCCACCTCAGGGGGGAAGCCGTGGCTTTCTATTCCTTTGCCCTCGGA
HiFi T2A for	CTCTGCTGACCTGTGGC
HiFi T2A_dB for	GCCCGGATTCTCCTCCACATCACCGCATGTTAGAAGAC
HiFi T2A_dB rev	GTCTTCTAACATGCGGTGATGTGGAGGAGAATCCGGGC
M13 rev	CAGGAAACAGCTATGAC
mamCas9 BglII rev_1	TGAAGATCTCTTGCAGATAGCAG
mamCas9 BglII rev_2	TTTAGATCTCTTGCAGATAGCAGATC
mamD10A mut for	ACAGCATCGGCCTGGCCATCGGCACCAACT
mamD10A mut rev	AGTTGGTGCCGATGGCCAGGCCGATGCTGT
MCS-3'PB XbaI for	ATATCTAGACGCTAGCTTTCAATTGATAGCGGCCGCATTGGATCCTTAGAATTCATAGGCGCGCCTAAAAGTTTTGTTACTTTATAGAAGAAATTTTGAG
mKO das 1 for	AAGGCCTGTCCTGGGAAAGGT
mKO das 2 rev	TCATTTGGCACTTGTGATTGC
mKO MluI for	ACTACGCGTCACAATGGCC
mKO2 AgeI for	TATACCGGTATGTTGAGTGTGATTAAACCAGAGATGAAG
mKO2 BsrGI-T2A rev	AAACTTGTACAGGGAATGAGCTACTGCATCTTCTACCTGC
mKO2 gen for	CCTCGAGATCCACCGGTATG
mKO2 gen rev	TGATAGGACCATCGGCAGGA
mKO2 MluI G67 corr	ACTACGCGTCACAATGGCCGAGGGCGGGCCAATGCCTTTCGCGTTTGACTTAGTGTCACACGTGTTCTGTTACGgCCACAGAGTA
P2A pTK EcoRI for	AAAGAATTCGAGCCCAAGAAGAAGAGGAAAGTCGGCTCCGGCGCTACCAATTTCTCCCTGCTCAAGCAAGCCGGAGACGTCGAGGAAAACCCAGGCCCAATGACCGAGTACAAGCCCAC
PEST puro rev	CGCAAGCGGCGGGGTGCCTGTCCATGCCGCTCTCCTGGGCGCAGCTCATGGGCAGGGTGCCATCGTCCTGAGCGGCCACCTCAGGGGGGAAGCCGTGGCTTTTTAGGCACCGGGCTTGCG
PEST T2A eGFP for	ATGGACAGGCACCCCGCCGCTTGCGCCAGCGCTAGGATCAACGTGGGTGAGGGCAGAGGAAGTCTTCTAACATGCGGTGACGTGGAGGAGAATCCGGGCCCTGTGGTGAGCAAGGGCGAG
pTK_dBamHI for	CGCCCCGCAACCTCCCCTTCTACGAGCGGCTCGGCTTCACCGTCACCGCCGACGTCGAGGTGCCCGAAGGACCGCGCACCTGGTGCATGACCCGCAAGCCCGGTGCCGGCTCCATGCCCA
pTK_dBsaI rev	GAAGGGGAGGTTGCGGGGCGCGGATGTCTCCAGG
puro BstEII for	GCGGGTCACCGAGCTG
puroR BstEII rev	CTCGGTGACCCGCTCG
SexAI-P2AG+2 for	AAAACCTGGTGCATGACCCGCAAGCCCGGTGCCGGCTCCGGAGAGGGCCGGGGCTCTCTGCTGACCTGTGGCGACGTGGAGGAGAACCCCGGCCCCTCCAAATGGTGAGCAAGGGCGAGG
SOD1 for #2	TGTGTAGACGTGAAGCCTTGTT
SOD1 rev #2	TGCTTTTTACTGCATCTTAGCAGAA
T7e	TAATACGACTCACTATAGGGC
TadA 7.10 XhoI rev	CACCTCGCGTTCATCTCG
TadA AgeI for_3	ACCGGTATGAGCGAGGTG
TadA_cc NotI for	TTTGCGGCCGCACCGGTATGAGCGAGGTGGAATTCAGCCATGAGTACTGGATGAGAC
VQR BamHI rev	CCTGGTGTCTCGCTGCCAGAGGATCCTCCGCTAGATCCTCCAGAGTCGCCTCCCAGCTG
VQR SanDI for	GATCGCCAGAAAGAAGGAC
xCas9 HiFi for	TCGCCAGAAAGAAGGACT
xCas9 HiFi rev	CTGGTGTCTCGCTGCC

Supplementary Table 2: direct cloning oligos

protospacer cloning oligos	
name	sequence
A2G_VQR g-bot (-)	aaacGGGTATACACGGCCTATCCC
A2G_VQR g-bot (+)	caccGGGATAGGCCGTGTATACCC
A2G_VQR g-top (-)	aaacTAACAGAACACGTGGGACAC
A2G_VQR g-top (+)	caccGTGTCCCACGTGTTCTGTTA
A2G_xC g0 (-)	aaacACCGGGTATACACGGCCTAC
A2G_xC g0 (+)	caccgTAGGCCGTGTATACCCGGT
gTLR_A2G +1 (-)	aaacCCGGGTATACACGGCCTATC
gTLR_A2G +1 (+)	caccGATAGGCCGTGTATACCCGG
gTLR_A2G +17 (-)	aaacTGTATACCCGGTGGTCGTAC
gTLR_A2G +17 (+)	caccGTACGACCACCGGGTATACA
gTLR_A2G +26 (-)	aaacGATAGGCCGTGTATACCCGC
gTLR_A2G +26 (+)	caccGCGGGTATACACGGCCTATC
gTLR_A2G +29 (-)	aaacCCGGATAGGCCGTGTATACC
gTLR_A2G +29 (+)	caccGGTATACACGGCCTATCCGG
gTLR_A2G +38 (-)	aaacGTATCTCCTCCGGATAGGCC
gTLR_A2G +38 (+)	caccGGCCTATCCGGAGGAGATAC
gTLR_A2G +4 (-)	aaacGGTATACACGGCCTATCCGC
gTLR_A2G +4 (+)	caccGCGGATAGGCCGTGTATACC
gTLR_A2G +8 (-)	aaacGGTGGTCGTAACAGAACACC
gTLR_A2G +8 (+)	caccGGTGTTCTGTTACGACCACC
gTLR_A2G -17 (-)	aaacCGTGTTCTGTTACGACCACC
gTLR_A2G -17 (+)	caccGGTGGTCGTAACAGAACACG
gTLR_dn -18 (-)	aaacACGTGTTCTGTTACGACCAC
gTLR_dn -18 (+)	caccGTGGTCGTAACAGAACACGT
gTLR_dn -26 (-)	aaacGGTGTCCCACGTGTTCTGTC
gTLR_dn -26 (+)	caccGACAGAACACGTGGGACACC
gTLR_dn -33 (-)	aaacTTGACCTGGTGTCCCACGTC
gTLR_dn -33 (+)	caccGACGTGGGACACCAGGTCAA
gTLR_dn -39 (-)	aaacTCGCCTTTGACCTGGTGTCC
gTLR_dn -39 (+)	caccGGACACCAGGTCAAAGGCGA
SOD1 g6 (-)	aaacCCAATGATGCAATGGTCTCC
SOD1 g6 (+)	caccGGAGACCATTGCATCATTGG
TLR g+66 (-)	aaacTCGGGAAATGCTTGTTTGAC
TLR g+66 (+)	caccGTCAAACAAGCATTTCCCGA
TLR g+76 (-)	aaacGGACAGGCCTTCGGGAAATC
TLR g+76 (+)	caccGATTTCCCGAAGGCCTGTCC

other cloning oligos	
name	sequence
BbsI-SacI-BbsI (+)	caccGGGTCTTCGTGGAGCTCATCGAAGACCT
BbsI-SacI-BbsI (-)	aaacAGGTCTTCGATGAGCTCCACGAAGACCC
BsaI-AhdI-BsaI (+)	gtgcAGAGACCGAATCGACCAGAGGTCTCG
BsaI-AhdI-BsaI (-)	aaaaCGAGACCTCTGGTCGATTCGGTCTCT
peg_scaff co (+)	GTTTTAGAGCTAGAAATAGCAAGTTAAAATAAGGCTA GTCCGTTATCAACTTGAAAAAGTGGCACCGAGTCG
peg_scaff co (-)	GCACCGACTCGGTGCCACTTTTTCAAGTTGATAACGG ACTAGCCTTATTTTAACTTGCTATTTCTAGCTCTA

RTt-PBS cloning oligos	
name	sequence
A2G_VQR peg-bot (-)	aaaaTAGGCCGTGTATACCCGATGGCCGTAACAGAAC
A2G_VQR peg-bot (+)	gtgcGTTCTGTTACGGCCATCGGGTATACACGGCCTA
A2G_VQR peg-top (-)	aaaaCCCACGTGTTCTGTTACGGCCACCGGGTATACA
A2G_VQR peg-top (+)	gtgcTGTATACCCGGTGGCCGTAACAGAACACGTGGG
A2G_xC g0 R15P13 (-)	aaaaGCCGTGTATACCCGGTGGCCGTAACAGA
A2G_xC g0 R15P13 (+)	gtgcTCTGTTACGGCCACCGGGTATACACGGC
A2G+1_R14P13 (-)	aaaaGGCCGTGTATACCCGGTGGCCGTAACA
A2G+1_R14P13 (+)	gtgcTGTTACGGCCACCGGGTATACACGGCC
A2G+1_R14P13_dP (-)	aaaaGGCCGTGTATACCCGGTGTCCGTAACA
A2G+1_R14P13_dP (+)	gtgcTGTTACGGACACCGGGTATACACGGCC
A2G+1_R14P15 (-)	aaaaTAGGCCGTGTATACCCGGTGGCCGTAACA
A2G+1_R14P15 (+)	gtgcTGTTACGGCCACCGGGTATACACGGCCTA
A2G+1_R14P8 (-)	aaaaTGTATACCCGGTGGCCGTAACA
A2G+1_R14P8 (+)	gtgcTGTTACGGCCACCGGGTATACA
A2G+1_R20P13 (-)	aaaaGGCCGTGTATACCCGGTGGCCGTAACAGAACAC
A2G+1_R20P13 (+)	gtgcGTGTTCTGTTACGGCCACCGGGTATACACGGCC
A2G+1_R20P15 (-)	aaaaTAGGCCGTGTATACCCGGTGGCCGTAACAGAACAC
A2G+1_R20P15 (+)	gtgcGTGTTCTGTTACGGCCACCGGGTATACACGGCCTA
A2G+1_R20P8 (-)	aaaaTGTATACCCGGTGGCCGTAACAGAACAC
A2G+1_R20P8 (+)	gtgcGTGTTCTGTTACGGCCACCGGGTATACA
A2G+4_R14P13 (-)	aaaaATAGGCCGTGTATACCCGGTGGCCGTA
A2G+4_R14P13 (+)	gtgcTACGGCCACCGGGTATACACGGCCTAT
A2G+4_R14P15 (-)	aaaaGGATAGGCCGTGTATACCCGGTGGCCGTA
A2G+4_R14P15 (+)	gtgcTACGGCCACCGGGTATACACGGCCTATCC
A2G+4_R14P8 (-)	aaaaCCGTGTATACCCGGTGGCCGTA
A2G+4_R14P8 (+)	gtgcTACGGCCACCGGGTATACACGG
A2G+4_R20P13 (-)	aaaaATAGGCCGTGTATACCCGGTGGCCGTAACAGAA
A2G+4_R20P13 (+)	gtgcTTCTGTTACGGCCACCGGGTATACACGGCCTAT
A2G+4_R20P13_dP (-)	aaaaATAGGCCGTGTATACCCGATGGCCGTAACAGAA
A2G+4_R20P13_dP (+)	gtgcTTCTGTTACGGCCATCGGGTATACACGGCCTAT
A2G+4_R20P15 (-)	aaaaGGATAGGCCGTGTATACCCGGTGGCCGTAACAGAA
A2G+4_R20P15 (+)	gtgcTTCTGTTACGGCCACCGGGTATACACGGCCTATCC
A2G+4_R20P8 (-)	aaaaCCGTGTATACCCGGTGGCCGTAACAGAA
A2G+4_R20P8 (+)	gtgcTTCTGTTACGGCCACCGGGTATACACGG
A2G+4_R20P8_dP (-)	aaaaCCGTGTATACCCGATGGCCGTAACAGAA
A2G+4_R20P8_dP (+)	gtgcTTCTGTTACGGCCATCGGGTATACACGG
SOD1 g6 R15P15 (-)	aaaaAGACCATTGCATCATTGGCCGCACACTGGT
SOD1 g6 R15P15 (+)	gtgcACCAGTGTGCGGCCAATGATGCAATGGTCT
SOD1 g6 R20P15 (-)	aaaaAGACCATTGCATCATTGGCCGCACACTGGTGGTAA
SOD1 g6 R20P15 (+)	gtgcTTACCACCAGTGTGCGGCCAATGATGCAATGGTCT

Supplementary Table 3: ssODNs for mKO2 gene correction

name	sequence
A2G+1+17_dP 100 (100-mer)	GCCAATGCCCTTCGCCTTTGACCTGGTGTCCCACGTGTTCTGTTACGGACACCGGGTATACACCGCCTATCCGGAGGAGATACC GGACTATTTCAAACAA
A2G+1+17_dP 150 (150-mer)	TACGCGTCACAATGGCCGAGGGCGGGCCAATGCCCTTCGCCTTTGACCTGGTGTCCCACGTGTTCTGTTACGGACACCGGGTA TACACCGCCTATCCGGAGGAGATACCGGACTATTTCAAACAAGCATTTCCCGAAGGCCTGTCCTGGG
A2G+1+17_dP 200 (200-mer)	TACGAGGGACATCAAGAGATGACACTACGCGTCACAATGGCCGAGGGCGGGCCAATGCCCTTCGCCTTTGACCTGGTGTCCC ACGTGTTCTGTTACGGACACCGGGTATACACCGCCTATCCGGAGGAGATACCGGACTATTTCAAACAAGCATTTCCCGAAGGC CTGTCCTGGGAAAGGTCGTTGGAGTTCGAAGATGG

Supplementary Table 4: qPCR primer and probes

name	sequence	5' modification	3' modification
PTPB2 for	TCTCCATTCCCTATGTTCATGC		
PTBP2 rev	GTTCCCGCAGAATGGTGAGGTG		
PTPB2 probe	ATGTTCCTCGGACCAACTTG	JOE	BHQ1
PB-PE qRT for	AGCGAATTCGAGCCCAAGAA		
PB-PE rev	GTGGGCTTGTACTCGGTCAT		
PB-PE probe	CGCTACCAATTTCTCCCTGCTCAAGCAAGCC	FAM	BHQ1

Supplementary Literature:

1. Sakaue-Sawano A*, et al.* Visualizing spatiotemporal dynamics of multicellular cell-cycle progression. *Cell* **132**, 487-498 (2008).

2. Eggenschwiler R*, et al.* A combined in silico and in vitro study on mouse Serpina1a antitrypsin-deficiency mutants. *Sci Rep* **9**, 7486 (2019).

3. Eggenschwiler R*, et al.* Improved bi-allelic modification of a transcriptionally silent locus in patient-derived iPSC by Cas9 nickase. *Sci Rep* **6**, 38198 (2016).

4. Chu VT*, et al.* Increasing the efficiency of homology-directed repair for CRISPR-Cas9-induced precise gene editing in mammalian cells. *Nat Biotechnol* **33**, 543-548 (2015).

5. Gaudelli NM*, et al.* Programmable base editing of A*T to G*C in genomic DNA without DNA cleavage. *Nature* **551**, 464-471 (2017).

6. Yusa K, Zhou L, Li MA, Bradley A, Craig NL. A hyperactive piggyBac transposase for mammalian applications. *Proc Natl Acad Sci U S A* **108**, 1531-1536 (2011).

7. Seo HW, Kim TM, Choi JW, Han BK, Song G, Han JY. Evaluation of combinatorial cis-regulatory elements for stable gene expression in chicken cells. *BMC Biotechnol* **10**, 69 (2010).

8. Li X*, et al.* piggyBac transposase tools for genome engineering. *Proc Natl Acad Sci U S A* **110**, E2279-2287 (2013).

9. Anzalone AV*, et al.* Search-and-replace genome editing without double-strand breaks or donor DNA. *Nature* **576**, 149-157 (2019).

10. Canver MC*, et al.* Variant-aware saturating mutagenesis using multiple Cas9 nucleases identifies regulatory elements at trait-associated loci. *Nat Genet* **49**, 625-634 (2017).
